# Cytogenetic Characterization and AFLP-Based Genetic Linkage Mapping for the Butterfly *Bicyclus anynana*, Covering All 28 Karyotyped Chromosomes

**DOI:** 10.1371/journal.pone.0003882

**Published:** 2008-12-08

**Authors:** Arjen E. Van't Hof, František Marec, Ilik J. Saccheri, Paul M. Brakefield, Bas J. Zwaan

**Affiliations:** 1 Department of Evolutionary Biology, Institute of Biology, Leiden University, Leiden, The Netherlands; 2 Faculty of Biological Sciences, University of South Bohemia, and Biology Centre, ASCR, Institute of Entomology, České Budějovice, Czech Republic; 3 School of Biological Sciences, University of Liverpool, Liverpool, United Kingdom; Texas A&M University, United States of America

## Abstract

**Background:**

The chromosome characteristics of the butterfly *Bicyclus anynana*, have received little attention, despite the scientific importance of this species. This study presents the characterization of chromosomes in this species by means of cytogenetic analysis and linkage mapping.

**Methodology/Principal Findings:**

Physical genomic features in the butterfly *B. anynana* were examined by karyotype analysis and construction of a linkage map. Lepidoptera possess a female heterogametic W-Z sex chromosome system. The WZ-bivalent in pachytene oocytes of *B. anynana* consists of an abnormally small, heterochromatic W-chromosome with the Z-chromosome wrapped around it. Accordingly, the W-body in interphase nuclei is much smaller than usual in Lepidoptera. This suggests an intermediate stage in the process of secondary loss of the W-chromosome to a ZZ/Z sex determination system. Two nucleoli are present in the pachytene stage associated with an autosome and the WZ-bivalent respectively. Chromosome counts confirmed a haploid number of n = 28. Linkage mapping had to take account of absence of crossing-over in females, and of our use of a full-sib crossing design. We developed a new method to determine and exclude the non-recombinant uninformative female inherited component in offspring. The linkage map was constructed using a novel approach that uses exclusively JOINMAP-software for Lepidoptera linkage mapping. This approach simplifies the mapping procedure, avoids over-estimation of mapping distance and increases the reliability of relative marker positions. A total of 347 AFLP markers, 9 microsatellites and one single-copy nuclear gene covered all 28 chromosomes, with a mapping distance of 1354 cM. Conserved synteny of *Tpi* on the Z-chromosome in Lepidoptera was confirmed for *B. anynana*. The results are discussed in relation to other mapping studies in Lepidoptera.

**Conclusions/Significance:**

This study adds to the knowledge of chromosome structure and evolution of an intensively studied organism. On a broader scale it provides an insight in Lepidoptera sex chromosome evolution and it proposes a simpler and more reliable method of linkage mapping than used for Lepidoptera to date.

## Introduction

The butterfly *Bicyclus anynana* (Nymphalidae, Satyrinae) is among the most extensively studied Lepidoptera species. It has been established as an emerging model organism to address many evolutionary questions with a particular focus on genetic and environmental effects on wing pattern formation [1]–[3], and on life history evolution and ageing [4]–[7]. Although this species has received much scientific attention, the physical features of its genome have yet to be described.

Lepidoptera chromosome numbers are usually between 28 to 32 pairs [8], [9], but can vary widely probably as a result of their holokinetic chromosome arrangement [10], [11]. The most striking examples at the genus level are found in *Agrodiaetus*, with haploid chromosome numbers that vary between 10 and 134 [10]. However, geographical intra-specific variability is also commonly observed in Lepidoptera [8], [12]. Geographical subspecies of the silk moth *Samia cynthia* show, besides different chromosome numbers, a high polymorphism of sex chromosomes [13], which may play a role in population and species divergence [14], [15]. An extraordinary variation in chromosome numbers, ranging from n = 12 to n = 88, was reported between populations of a *Philaethria dido* species complex, which is no longer regarded as single species, since no evidence of hybrids between individuals of sympatric populations with different chromosome numbers was found [16], [17]. The karyotype variation within the genus *Bicyclus* is less spectacular (Supplement S1). With the exception of *B. auricrudus* that has a reported haploid chromosome number of 14, all karyotyped species have between 26 and 29 pairs, with n = 28 being the predominant count [18]–[20]. However, geographical within-species variation has been observed in *B. funebris* with n = 28 in Uganda and n = 29 in Senegal [18], [19]. A haploid chromosome number of 28 was reported in *B. anynana* from Entebbe, Uganda [18], but given the geographical variability in Lepidoptera there is need for confirmation since the material used in the present study originates from Nkhata Bay in Malawi, about 1300 km to the south.

Identification of individual chromosomes based on size and banding patterns is difficult in Lepidoptera because of the large number of small and equally sized chromosomes that are not susceptible to banding techniques during mitosis. Much longer meiotic chromosomes in the pachytene stage provide better resolution, but their chromomere patterns are usually not fully distinctive [11], [21]. In addition, lepidopteran chromosomes are holokinetic, i.e. they lack a distinct primary constriction (the centromere) and spindle microtubules are attached to a large kinetochore plate, which covers significant part of the chromosome surface [22]. Thus, the chromosomes cannot be distinguished or characterized by centromere position. The most useful visual characteristics to distinguish lepidopteran chromosomes are the presence of nucleolar organising regions (NORs) associated with nucleoli and heterochromatin of the W chromosome in the sex-chromosome (WZ) pachytene bivalents of females. However, this accounts only for a small fraction of the chromosomes [23], [24].

Despite the abundance of lepidopteran species and their economical relevance, linkage maps are currently available for only six species. One reason for this is that the generally large number of chromosomes in this taxon requires a relatively large number of markers to cover all chromosomes with sufficient density. Additionally, a substantial part of the polymorphisms in the offspring cannot be used for positional mapping since the maternally transmitted markers are non-recombinant in Lepidoptera. The maternally transmitted markers obscure a large part of the paternally transmitted genotypes when using dominant markers, resulting in an even greater loss of information [25], [26]. The most detailed linkage information in Lepidoptera comes from the domesticated silkworm *Bombyx mori*, for which a number of linkage maps have been constructed based on RAPD [27], [28], RFLP [29], AFLP [30], microsatellites [31], and BAC sequences [32]–[34]. In addition, all genetic linkage groups (LGs) were successfully assigned to individual chromosomes in this species [35]. The other lepidopteran linkage maps have been constructed for *Heliconius melpomene* [25], *H. erato* [26], [36], *Colias eurytheme-C. philodice* hybrid [37], *Ostrinia nubilalis* [38] and *Plutella xylostella* [39] based on RFLP, AFLP, microsatellites, allozymes and single copy nuclear genes.

When using a cross with dominant markers such as AFLP's, the general approach in Lepidoptera mapping procedures is to divide the offspring marker data into three groups based on the F_1_ marker genotypes. Markers that are heterozygous in both F_1_ parents segregate in the F_2_ with a 3∶1 Mendelian ratio. Markers that are recessive homozygous in the F_1_ male and heterozygous in the F_1_ female have a 1∶1 ratio in the F_2_ offspring. These markers are used for LG assignment and for identification and exclusion of the uninformative female-inherited component in the 3∶1 markers. The markers that are recessive homozygous in the F_1_ female and heterozygous in the F_1_ male also have a 1∶1 ratio in the offspring. These markers, combined with the male-inherited component of the 3∶1 marker genotypes, are used for constructing the final linkage map [25], [26], [36].

When using only the 3∶1 markers, the outcome is a linkage map with two LGs per chromosome (2n LGs). The two sets of homologous LGs are incompatible and can only be combined with anchoring markers. Male informative markers, allelic AFLPs and microsatellites can act as such anchors and there are various approaches to integrate the two sets of dominant markers. For example, Lepidoptera specific software was designed to create a linkage map for *B. mori* because it was argued that MAPMAKER 3.0 [40] is unsuitable for this purpose [29]. In other studies, the final step is performed with MAPMAKER 3.0, allthough in some cases the preceding steps were done in JOINMAP 3.0 [41] or specifically designed programs [25], [26], [36], [39]. Alternatively, the LGs in repulsion were presented as two different sets [27], [28], or one integrated set that was based on the average distances of anchoring markers [34].

Here we report on a novel approach for the final step in Lepidoptera linkage maping by using the option in JOINMAP to join maps, i.e. to present the two opposite phased homologous maps as different mapping populations and use the software to integrate them based on the anchoring markers. The advantages are that the female-derived component can be removed instead of presented as missing data, and the same software combines the two phases automatically. To compare our mapping distance with that of other species of butterfly, we also performed a MAPMAKER analysis because Mapping distances generated by the two programs can differ substantially [42], [43]. In general, these differences are caused by the different algorithms that are used. MAPMAKER determines the mapping distance based on maximum likelihood multipoint estimates, while JOINMAP uses linear regression of pairwise distances. Additionally, when using dominant markers in species with only one recombining sex, the manner in which the uninformative part of the data are treated also has an effect on mapping distance.

## Methods

### Cytogenetic procedures

Spread preparations of pachytene oocytes were obtained following the protocol in [44] for pachytene mapping. Ovaries of 5^th^ instar larvae were dissected in physiological solution, then fixed for 20 min in Carnoỳs fixative (6 : 3 : 1 ethanol-chloroform-acetic acid), macerated in 60% acetic acid, spread on a slide at 45°C, dehydrated by three washes in increasing concentrations of ethanol (70%, 80%, and 96%, 30s each), and dried at room temperature, leaving the preparations suitable for different types of staining. Some preparations were stained for 5 min and mounted in 2.5% lactic acetic orcein. Others were stained with YOYO-1 fluorescent dye (Molecular Probes Inc., Eugene, OR, USA) under the following conditions: the dry preparations were first soaked for 5 min in PBS (phosphate buffered saline), then stained with 50 µl of 100 nM YOYO-1 in PBS for 20 min, briefly washed in tap water, air-dried and mounted in 20 µl of antifade based on DABCO (1,4-diazabicyclo(2.2.2)-octane; Sigma-Aldrich, St. Louis, MO, USA) (for details, see [45]).

Male metaphase I and II chromosomes were obtained from testes of the 5^th^ instar larvae. The testes were dissected in physiological solution, pretreated in hypotonic solution (0.075M KCl) for 15 min, and then fixed in Carnoy's fixative for 15 minutes. The testes were subsequently squashed in 20 µl of 50% acetic acid using a siliconised cover slip, followed by dehydration in an alcohol series as described above. Staining involved a 5 min incubation in PBS/1% Triton-X, followed by 15 min in PBS/1% Triton-X with 0.25 µg/ml DAPI (4′,6-diamino-2-phenylindole; Sigma-Aldrich). The slides were then rinsed for 5 min in PBS/1% Triton-X with 1% Kodak PHOTO-FLO, followed by 10s rinsing in H_2_O containing 1% Kodak PHOTO-FLO. Finally, the preparations were mounted in 20 µl of antifade.

To determine the sex chromatin status (see [46]), preparations of polyploid nuclei were made from Malpighian tubules of 5^th^ instar larvae. The tubules were dissected in physiological solution, fixed in Carnoy's fixative for 2 min, and then stained with 1.5% lactic acetic orcein for 4 min.

### Linkage analysis and map construction

#### Cross design

The linkage analysis was based on a cross between individuals from divergent selection lines for eyespot size on the ventral hindwing, designated High (H) and Low (L) for large and small eyespots respectively [3], [47]. An H-female was mated to an L-male (P generation), and subsequently, 15 full-sib F_1_ crosses were set up by combining random brothers and sisters to produce segregating F_2_ offspring. The larvae were raised on maize plants and the adults were fed with banana. They were reared at 23°C to minimize the effect of temperature on eyespot size, since this temperature is an intermediate between the temperature that would produce small (20°C) and large (27°C) eyespots as a result of phenotypic plasticity. The cross that produced the largest amount of F_2_ adults was selected to produce the linkage map. All procedures have been performed following our institutional animal husbandry guidelines. From a total offspring of 71 males and 113 females, 23 individuals from both ends of the phenotypic extremes of the F_2_ generation were genotyped in each sex (i.e. 92 F_2_ individuals in total). DNA was extracted from half a thorax using DNeasy tissue spin columns (Qiagen GmbH, Hilden, Germany).

#### AFLP

We followed a modified procedure of the AFLP technique [48]. Digestion and ligation were performed simultaneously for two hours at 37°C in 25 µl 1×T4 ligase buffer containing 1.2 units of both *Mse*I and *Eco*RI (NEB, Ipswich, MA, USA), 0.612 µM Mse-adapter (5′-GACGATGAGTCCTGAG-3′+5′-TACTCAGGACTCAT-3′), 0.068 µM Eco-adapter (5′-CTCGTAGACTGCGTACC-3′+5-AATTGGTACGCAGTCTAC-3′), 0.6 Weiss units T4 Ligase, 2.5 µg BSA and 5 µl DNA extract from the 2^nd^ Qiagen DNeasy tissue kit elution (corresponding to approximately 125 ng DNA).

Preamplification was performed in 15 µl 1×AFLP Amplification Core Mix Module (Applied Biosystems, Foster City, CA, USA) supplied with 0.12 µM Eco+A primer (5′-GACTGCGTACCAATTCA-3′), 0.92 µM Mse+C primer (5′-GATGAGTCCTGAGTAAC-3′), and 2 µl undiluted restriction-ligation product as template. Preamplification PCR cycle was 120s 72°C, 120s 94°C, followed by 20 cycles of 10 s 94°C, 30 s 56°C, 120 s 72°C.

Selective amplifications with 33 different primer combinations were processed in 10 µl 1×Core Mix with 0.05 µM fluorescently labeled Eco+ANN primer, 0.25 µM Mse+CNN primer and 1 µl 10×diluted preamplified product as template. For sequence and fluorescent labels of the primers see Table 1. Amplification was performed with 60 s 94°C, then 9 cycles of 10 s 94°C, 30 s T_a_ (annealing temperature), 120 s 72°C, with T_a_ decreasing 1°C per cycle from 65°C down to 57°C. Then 25 cycles of 10 s 94°C, 30 s 56°C, 120 s 72°C, and a final extension of 30 min at 72°C. Twelve of the combinations were genotyped on an ABI 377 automated sequencer with 3 different dyes and ROX500 size standard, and an additional 21 on an ABI 3100 with 4 dyes and LIZ500 size standard. The ABI377 data output was analyzed with GENOGRAPHER 1.6.0 [49] and the ABI3100 generated data with GENOTYPER 3.6. (Applied Biosystems). We use the term “peakpresent” to indicate an AFLP amplicon that shows up as a peak on capillary fragment analysis systems and which is either homozygous or heterozygous and “peakabsent” for the recessive homozygote.

**Table 1:** AFLP primer combinations and fluorescent dyes.

*Mse*I-based primer	*Eco*RI-based primer blue	*Eco*RI-based primer green	*Eco*RI-based primer yellow	*Eco*RI-based primer red	Instrument
mCAA	eACA 5-FAM	eACC JOE	eAAC NED	not used	ABI 377
mCAC	eACA 5-FAM	eACC JOE	eAAC NED	not used	ABI 377
mCAT	eACA 5-FAM	eACC JOE	eAAC NED	not used	ABI 377
mCGC	eACA 5-FAM	eACC JOE	eAAC NED	not used	ABI 377
mCAG	eACA 6-FAM	eAAC VIC	eACC NED	not used	ABI 3100
mCGA	eACA 6-FAM	eAAC VIC	eACC NED	not used	ABI 3100
mCGG	eACA 6-FAM	eAAC VIC	eACC NED	eACG PET	ABI 3100
mCGT	eACA 6-FAM	eAAC VIC	not used	eACG PET	ABI 3100
mCTC	eACA 6-FAM	eAAC VIC	eACC NED	eACG PET	ABI 3100
mCTG	eACA 6-FAM	eAAC VIC	eACC NED	eACG PET	ABI 3100

The first column contains the different *Mse*I-based primers used. The next four columns contain the fluorescently labeled *Eco*RI-based primers that were used in combination with the *Mse*I-based primer within the same row. The primers are 19 bp in length and consist of a 16 bp core sequence and a 3 bp extension. “m” is short for a GATGAGTCCTGAGTAA core sequence and “e” stands for a GACTGCGTACCAATTC core sequence. “m” and “e” are followed by the three base extensions that differentiate them. The colors of the fluorescent labels of the *Eco*RI-based primers are presented in the column headers, and the fluorescent 5′ modifications in the cells below them (5-FAM, 6-FAM, JOE, VIC, NED and PET). Individual AFLP markers in Fig. 2 & 3 are characterized by the eNNN-mNNN combinations shown in this table and the PCR product size. The final column describes which fragment analysis instrument was used.

#### Microsatellites

The microsatellite markers available for this species were processed under the conditions described in [50], except in this case they were amplified with NED, PET, 6-FAM or HEX modified fluorescent primers, run with LIZ-500 size standard on an ABI 3700 fragment analysis instrument and analysed with Genotyper 3.6 (primers, size standard, software and ABI 3700 from Applied Biosystems).

#### *Tpi* genotyping

RNA was extracted from ground thorax with TRIZOL (Invitrogen, Carlsbad, CA, USA) following the methods suggested by the manufacturer. cDNA was synthesized with SUPERSCRIPT III (Invitrogen) with 50 ng template and a T_17_ primer under standard conditions. A section of the *Tpi* (Triose-phosphate isomerase) gene was amplified with arthropod-specific degenerate primers *197fin*1F and *197fin*2R [51]. PCR was performed in 1×Amplitaq PCR buffer I, 0.6 units Amplitaq Gold polymerase (buffer and polymerase supplied by Applied Biosystems) 0.4 µM of each primer, 0.2 µM dNTP and 1 µl of the cDNA in a final volume of 20 µl. The PCR cycle was 9 min 94°C, then 35 cycles of 30 s 94°C, 30 s 50°C, 45 s 72°C. The PCR product was purified with EXOSAP-IT (Amersham plc, Little Chalfont, UK), sequenced with the BigDye 3.1 kit (Applied Biosystems), and analyzed on an ABI 3100 sequencer (Applied Biosystems). Gene-specific primers Ba_TPI_207U (TTCGGCTGAGATGATAAAGG) and Ba_TPI_473L (AGTACCAATGGCCCACACTG) were designed within the *Tpi* sequence to amplify an intronic region, using the same genomic template as for the AFLP reactions. PCR conditions were as described above, except for using T_a_ 52°C instead of 50°C. The F_1_ parents were screened for SNPs (single nucleotide polymorphisms) by means of sequencing the intron. Genotyping the F_2_ offspring was based on PCR amplification (as above), the amplicons were subsequently treated with 1 unit of *Alu*I restriction enzyme (NEB) for 2 h at 37°C, which either cuts a 230 bp fragment into 30 bp and 200 bp or leaves it intact, depending on the genotype. The restriction pattern was screened on a 3% agarose gel. The *Tpi* partial cds and intron sequence are submitted to GENBANK under accession numbers EU675861 and EU675862.

#### Data sorting into FI, MI, BI, and sex-linked markers

The AFLP markers were divided into different groups, depending on the F_1_ genotypes. Female informative (FI) markers are present in the F_1_ female and absent in the F_1_ male and segregate 1∶1, male informative (MI) markers are present in the F_1_ male and absent in the F_1_ female and segregate 1∶1 as well. BI (both informative) markers segregate with a 3∶1 ratio, resulting from F_1_ male and female that are both heterozygous peakpresent. Z-linked markers were identified by a peakpresent in all male offspring and a 1∶1 ratio in the female offspring (representing an F_1_ WZ**^+^** (♀)×Z**^+^**Z**^−^** (♂) F_1_ cross, with “+” = peakpresent allele and “–” = peakabsent allele). All F_2_ female MI-markers were compared with this Z-specific 1∶1 pattern in JOINMAP to reveal the WZ**^−^**×Z**^+^**Z**^−^** crosses in which both male and female offspring have a 1∶1 ratio.

#### Identification of chromosome prints

Due to the absence of meiotic recombination in females, syntenic FI-markers are transmitted to the offspring in complete association, independent of their relative position. Consequently, they cannot be positioned within LGs. A cluster of syntenic FI-markers displays a chromosome-specific pattern of F_2_ genotypes, which is identical for all loci on the same chromosome and which displays the exact opposite pattern in all markers in repulsion. This fixed set of genotypes has been named the “chromosome print” [28]. The number of chromosome prints per individual equals the haploid autosomal chromosome number. Their identification in *B. anynana* was carried out as described in [25], by grouping the FI-markers together with JOINMAP under strict conditions (LOD >8), allowing just a small number of genotyping errors. The linkage phase describes on which parental (F_1_) chromosome the peakpresent of a marker lies and from which grandparent (P-generation) it came. If the marker is present in the grandmother and absent in the grandfather, the linkage phase is “0”, the reverse gives linkage phase “1”. When the marker is present in both grandparents, the linkage phase is determined by the software based on co-segregation in the F_2_ with markers for which linkage phases are known. Linkage phases consist of a maternal and a paternal component, indicating marker orientation (and P-origin) in the F_1_ mother and the F_1_ father.

The chromosome prints were numbered based on the output order of the software. It is important to reduce the number of errors in chromosome prints to a minimum because they are subsequently used for error detection and identification and removal of uninformative markers. With multiple FI-markers defining a chromosome print, inconsistencies were rescored and when persistent, the chromosome print was based on the most common genotype in the inconsistent individual.

Chromosome prints for chromosomes without FI-markers were reconstructed based on BI and MI-markers as described in Supplement S2. This was done after the LG assignment described below. In addition, ten LGs with available (FI-based) chromosome prints were also reconstructed in this way to validate the reconstruction technique.

#### BI and microsatellite linkage group assignment

BI and microsatellite markers were grouped by screening them against the 21 chromosome print patterns in JOINMAP with a LOD threshold of 3. This mapping step also established the linkage phase of the markers. Markers in the six LGs for which chromosome prints were initially not available were assigned to LG22-LG27.

The markers were subsequently screened by a “forbidden genotype” analysis to confirm or reject correct LG assignment and to detect scoring errors [29], [39]. This procedure is based on the fact that certain marker combinations within an individual cannot occur because it would involve recombination in females. This screening procedure is explained in more detail in Supplement S3. The threshold to exclude markers from further analysis was set to three or more forbidden genotypes.

#### Identification of allelic (codominant) AFLPs

Part of the observed variation in AFLP data is caused by indels (insertions or deletions) between the two restriction sites at a single locus, resulting in amplicons of different sizes. To determine whether two BI loci are in fact different alleles of the same locus, we applied the following criteria: (1) they must have the same primer combination; (2) they must group together in the same LG when presented as independent loci in the initial uncensored BI screening; and (3) linkage phases of markers with 3∶1 ratio must be opposite for both the maternal and the paternal component. Either one or both peaks present in an individual would be a prerequisite for codominance in species with recombination in both sexes, but with non-recombining females, that same condition is already covered by forbidden genotype restrictions.

MI alleles were detected as well, but they do not provide more analytical power when combined together into one codominant marker as is the case in the BI-markers. Their opposite paternal linkage phases produce fully complementary peak patterns that hold the same mapping information.

#### Censoring of female-derived BI-markers in the F_2_

The BI-markers (with a 3∶1 ratio in the offspring) obtain half their peakpresent alleles from the F_1_ mother and the other half from the F_1_ father. A female derived peakpresent obscures the male-derived allele in dominant markers, so that it is impossible to distinguish between F_2_ homozygotes and heterozygotes. This is not an issue when mapping species with recombination in both sexes, because mapping software can treat these unknown allele combinations as “either heterozygous or homozygous”. However, without recombination in the females, genotype scores that have a positive F_1_ female signal have to be excluded from analysis, which means that part of the paternal information is also lost. What remains are scores for those individuals that obtained a peakabsent from the female and either a present or absent from the male in a 1∶1 ratio. The criteria for filtering out the female component is straightforward because the female BI peakpresent is always fully linked to either a positive or negative chromosome print value, depending on their relative maternal linkage phases (Supplement S4). Markers from individuals with a positive chromosome print value must be removed when they have the same maternal linkage phase as the chromosome print, and markers in repulsion with the chromosome print must be removed in the remainder of the individuals.

#### Assignment of linkage groups for MI-markers

The censored BI genotypes are initially replaced with “missing data”. The BI and microsatellite markers with their LG designations are then analyzed together with the MI and microsatellite markers in JOINMAP to establish to which LGs they belong.

#### Final map construction

Microsatellites were translated to their male informative component as described in Supplement S5, resulting in MI-markers with a 1∶1 ratio. These were then combined with the MI- and censored BI-markers for each separate chromosome. Each chromosome set was then divided in two subsets, based on their chromosome print values (Supplement S4). The BI markers in these two subsets are of opposite maternal linkage phase as a result of the exclusion of the censored BI genotypes. All the subsets were individually presented to JOINMAP for linkage map construction. Subsequently, the sets of linkage maps representing the same chromosomes with suitable anchoring markers are combined with the “Combine groups for map integration” command in JOINMAP. The remaining sets (without anchoring markers) remain as separate LGs. The integration of the two subsets is represented schematically in Supplement S4.

The Z chromosome markers were divided into male- and female F_2_ offspring. The female F_2_ offspring have a 1∶1 ratio for all markers, while the F_2_ males have 100% peakpresent when the F_1_ female is also peakpresent. These 100% male scores were excluded from analysis and all the female markers and the remaining male markers were separately mapped and then joined as described above.

#### Comparison between JOINMAP and MAPMAKER

Besides the linkage map construction with JOINMAP, we followed the procedures described in [26] for constructing a linkage map with MAPMAKER 3.0.

All steps except the “Final map construction” were identical to the procedures described above, since [26] used JOINMAP for that part of the analysis. The main difference from the JOINMAP approach in this final step is that the censored BI-markers were replaced by “missing data” rather than excluded, and that the markers belonging to the same LGs were analysed together instead of in two separate groups. For LGs without sufficient anchoring markers, the subgroups with the largest mapping distance were compared.

## Results

### Cytogenetics

#### Chromosome number

**Figure 1:**
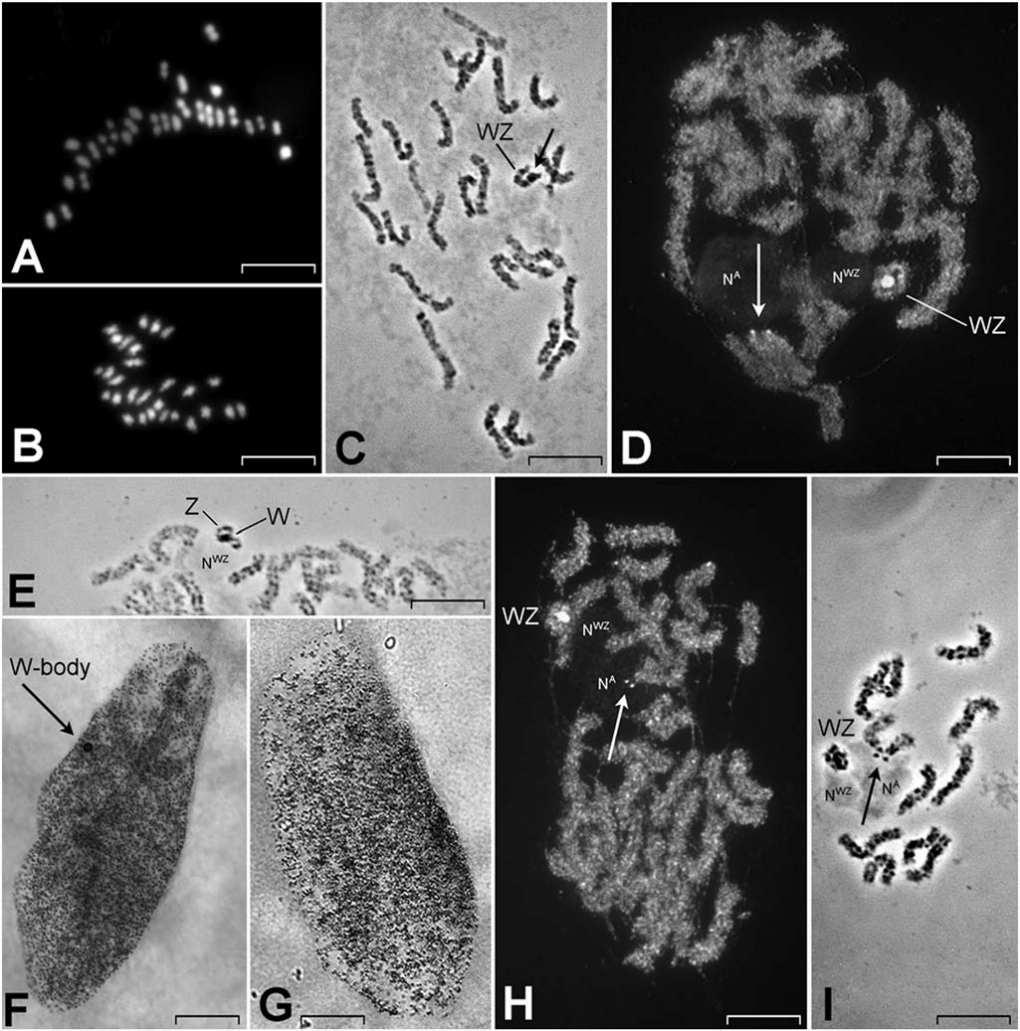
Preparations of meiotic cells and somatic interphase nuclei in *Bicyclus anynana*. (A) Squashed DAPI-stained male metaphase I bivalents; (B) squashed DAPI-stained male metaphase II chromosomes; (C) spread orcein-stained female pachytene complement showing chromomere patterns; note the small heterochromatic W chromosome associated with the terminal segment of the Z chromosome (arrow); (D) spread YOYO-1-stained female postpachytene complement showing a curious WZ bivalent, in which the Z chromosome strand is wrapped around the body-like W chromosome, and two nucleoli, one associated with an autosome bivalent (N^A^) and the other with the WZ bivalent (N^WZ^); note small heterochromatin dots (arrow) highlighted with YOYO-1 at the end of each chromosome of the NOR-autosome bivalent; (E) orcein-stained female pachytene spread, showing a WZ-bivalent where the W chromosome is associated with the central part of the Z chromosome; (F) a polyploid nucleus of the female Malpighian tubule cell showing a small sex-chromatin body (arrow), representing multiple copies of the tiny W chromosome; (G) a polyploid nucleus of the male Malpighian tubule cell without sex chromatin; (H) YOYO-1 stained female pachytene spread showing the NOR as stalked dots (arrow) in the nucleolus; (I) orcein-stained female pachytene spread with two conspicuous chromomeres (arrow) within the nucleolus. Scale bars indicate 10 µm in (A-D and H,I) and 50 µm in (F, G).

The analysis of metaphase I bivalents and male metaphase II chromosomes in male meiosis, and pachytene bivalents in female meiotic prophase I showed a haploid chromosome number of 28 for *B. anynana* in our stock from Malawi (Fig. 1A–C). This is consistent with the findings of [18] for *B. anynana* from Uganda and thus, there is no evidence for geographical variation in chromosome numbers in this species. Orcein staining of pachytene bivalents provided the characteristic chromomere pattern that differentiated the chromosomes to a certain level (Fig. 1C). However, we did not assign chromosome numbers based on these patterns since it is not clear with which linkage groups they correspond.

#### Sex chromosomes

Male pachytene spreads displayed 28 bivalents per nucleus that were aligned over their full length. Female pachytene oocytes showed 27 fully-paired bivalents and a pair of sex chromosomes, consisting of a small heterochromatic W chromosome that has a circular arrangement and a Z chromosome that was wrapped around it in the majority of nuclei (Fig 1D); in some nuclei, the W chromosome was associated with a terminal segment of the Z chromosome (Fig. 1C) or less often with a central part of the Z chromosome (Fig. 1E) and formed a short thick rod or a body-like structure. A comparison of the male and female chromosome complements shows that *B. anynana* has a WZ/ZZ (female/male) sex chromosome system, typical for the majority of advanced Lepidoptera (reviewed in [11]).

Large, highly polyploid interphase nuclei of the Malpighian tubules do not form lobes as is seen in some Lepidoptera (cf. [52]), but have oval shapes. In females, each nucleus showed a small heterochromatin W-body (i.e. sex chromatin) that was absent in males (Fig. 1F,G). The small size of the W-body was consistent with the tiny W chromosome observed in pachytene oocytes.

#### Nucleolar organising regions

Two distinct nucleoli were regularly observed in YOYO-1-stained pachytene spreads. One was associated with an autosome bivalent, the other with the WZ bivalent (Fig. 1D,H,I). The association with the WZ bivalent is not apparent in Fig. 1D and 1H since the nucleolus also borders autosomal bivalents, but it was consistent in all examined nuclei. At the end of the autosome bivalent, a pair of YOYO-1-positive dots was immersed into the nucleolus mass. The dots probably composed of heterochromatin were often separated from the main chromosome bodies by a constriction, obviously representing the nucleolus organizing region (NOR) (Fig 1H). In orcein-stained pachytenes, two conspicuous chromomeres were seen at the end of this NOR-bivalent (Fig 1I). These chromomeres most likely correspond with the two heterochromatic dots highlighted with YOYO-1 (Fig. 1D,H).

### Linkage mapping

#### Genetic markers

A total number of 458 polymorphic segregating markers was generated with AFLPs. The effective number was smaller because the female informative markers do not contribute to mapping, a small number of markers failed the forbidden genotype screening, and 52 markers that behaved as alleles were merged to form 26 single locus codominant markers. This resulted in 347 AFLP loci that could be used for the construction of the linkage map. The markers cover all chromosomes except for the W chromosome, which cannot be mapped even if markers were available because this chromosome not involved in recombination. Additionally, there were seven polymorphic microsatellites that could be positioned on the map and another two that could only be assigned to specific LG's by their female informative component because they were homozygous in the F_1_ male. This number is far lower than the number of microsatellite loci available for *B. anynana* because many were not informative in the P-generation to start with, and other loci inherited an uninformative set of alleles from the P-generation to the F_1_ due to the bottleneck conditions of the full-sib cross design. The *Alu*I digestion of the genomic *Tpi* amplicons gave a restriction pattern in male F_2_ offspring of either a 230 bp fragment, a 200 bp (and a 30 bp) fragment, or both of them within the same individual, thus representing both homozygotes and the heterozygote. Female F_2_ offspring had either the 230 bp or the 200 bp fragment (but not both) per individual, thereby showing a hemizygous (Z-linked) pattern.

Chromosome prints based on FI-markers were available for 21 of the 27 autosomes, another three were reconstructed from BI and MI-markers (LGs 22, 25, 27) and the remaining three were based on BI-markers alone (LGs 23, 24, 26), with random 1∶1 designation for the unassigned values as described in Supplement S2. The empirical verification of the BI+MI based reconstruction for LGs with chromosome prints already available gave an exact match between “chromosome print” and “reconstructed chromosome print” in eight out of 10 cases, one with a single error and one with three, giving a total of only four inconsistent values out of 920. The verification of difference between the BI-only reconstructed maps and the actual maps (performed on the same 10 control LGs) showed a deviation of 2 cM at most for the entire mapping distance, and the markers always remained in the same order.

**Figure 2:**
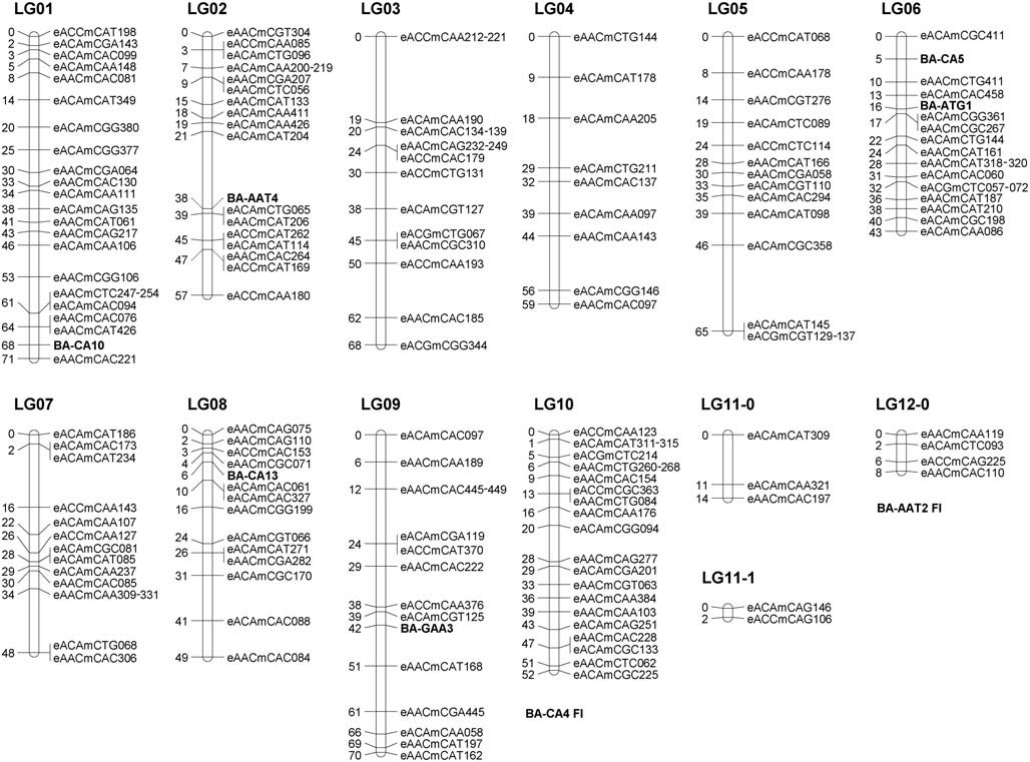
Linkage map of LG1-12. Vertical bars represent chromosomes and show the mapping distance in centimorgan (cM) on the left and the corresponding markers on the right. Microsatellites are displayed in bold and start with “BA”, the two microsatellites with only FI polymorphism are placed underneath the LG's they belong to. AFLPs are named according to their selective primer extension and amplicon size. The “e” stands for the fluorescent *Eco*RI-based primer and the “m” stands for the non-fluorescent *Mse*I-based primer. AFLPs with two amplicon sizes per primer combination (e.g. eACCmCAA212-221 in LG03) are codominant. A vertical line indicates that markers are less than 1 cM apart (e.g. eACAmCGA119 and eAACmCAT370 in LG09).

**Figure 3:**
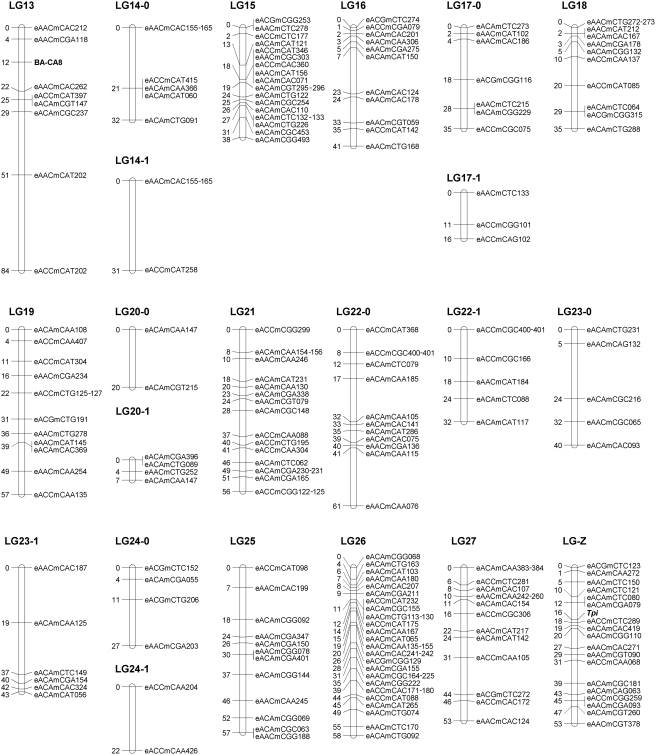
Linkage map of LG13-27 and Z.

The final linkage map is shown in Fig. 2 and 3. Twenty chromosomes had sufficient anchoring markers to create integrated LGs following the procedures outlined in Supplement S4. Eight chromosomes had either one or no anchoring markers (chromosomes 11, 12, 14, 17, 20, 22, 23, 24), which prevented integration. These are represented in Fig. 2 and 3 as separate linkage groups per chromosome with unknown position and orientation relative to each other. These two subsets represent markers available from the high and low eyespot selection lines respectively. The Z chromosome contains 18 evenly dispersed markers and the *Tpi* gene. The mapping lengths of the chromosomes range from 8 to 84 cM, but we assume that the smaller linkage groups have insufficient coverage rather than representing chromosomes that are relatively small. Therefore, the estimated map length does not necessarily reflect the actual chromosome length.

#### Comparing mapping procedures; JOINMAP with separate phase analysis vs. MAPMAKER with missing data censoring

The mapping order in MAPMAKER was similar to the JOINMAP output for most chromosomes. However, in some LG's with low proportions of anchoring markers vs. BI markers, or unevenly distributed anchoring markers, large rearrangements sometimes occurred. This is caused by the fact that MAPMAKER compares small subsets of markers rather than all representatives of an LG at the same time. MAPMAKER initially uses a maximum of eight markers, and subsequently positions additional markers within the initial (eight marker) map. Finally, the mapping order is fine-tuned by using a sliding window of five markers (ripple command). The use of a subset of markers (i.e. eight initial markers or five ripple markers) that is made up of BI markers of both maternal linkage phases and less than two anchoring markers, results in an unreliable suggested marker order. The reliability of the initial (eight marker) map can be improved by including all available MI and codominant markers, but with the ripple command the representative markers cannot be hand-picked because their grouping depends on the provisional marker order suggested by MAPMAKER. Similar to the ripple command that is used to determine marker order, a sliding window analysis also reveals the reliability of the marker order, by comparing the likelihood of the most likely marker order with alternative orders (flips test). This test is confronted with the same bi-phasic incompatibility problems and cannot be used on a censored data set with missing data. The consequences of comparing only subsets of markers within a linkage group (i.e. sliding window) are illustrated with an example based on LG21, which is characterized by codominant anchoring markers close to both ends and ten dominant markers of both phases in between them (Supplement S6). JOINMAP also performs a ripple test, which is based on a sliding window of only three markers. With “missing data” input, this results in even more problems than in MAPMAKER because the chance that two anchoring markers are included in a subset of just three markers is far smaller than in a subset of five. This is presumably the reason why for the final mapping step in some butterfly linkage maps JOINMAP has been replaced by MAPMAKER.

The marker order suggested by JOINMAP (following the procedures used for the present linkage map) is far more reliable than the MAPMAKER approach because it does not attempt to map incompatible BI markers relative to each other directly. The ripple test, which can cause serious problems with missing data analysis, strongly increases the reliability of the marker order when analyzing markers of each maternal linkage phase separately in JOINMAP. Instead of reporting a flips test value, JOINMAP simply excludes markers that do not meet the criteria for reliable neighboring markers (recombination frequency smaller than 0.4 and LOD larger than 1.0). MAPMAKER on the other hand always suggests a mapping order and will always produce a linkage map that includes all presented markers.

The mapping distances given by MAPMAKER were larger than those produced by JOINMAP under all circumstances. The mapping distances decreased substantially with error detection activated in MAPMAKER, but were on average still 38% larger than in JOINMAP, ranging from 1.02 to 2.14 times in size for the different LGs (Supplement S7). The total mapping distances are 1873 vs. 1354 cM for MAPMAKER and JOINMAP respectively. The data are presented in different ways to each program, with the censored BI-markers as missing data in MAPMAKER and excluded in JOINMAP. Since JOINMAP has difficulties with high proportions of non-overlapping missing data, a comparison with identical data input was not possible for the MI-markers combined with censored BI-markers. Therefore, the software was also compared based only on MI-markers, thus avoiding censoring of markers. Fourteen LGs had sufficient MI-markers to construct linkage maps with MAPMAKER again giving higher values than JOINMAP, but now with only 17% difference. The genome size of *B. anynana* is 0.49 pg [53], which corresponds with approximately 480 Mb [54]. This means that the JOINMAP based linkage map is 355 Kb/cM and the MAPMAKER based map 256 Mb/cM.

## Discussion

### Cytogenetics

The cytogenetic characteristics combined with the inheritance patterns of genetic markers of *B. anynana* correspond to those generally found in Lepidoptera. Female heterogamety is confirmed by the presence of a WZ bivalent in pachytene oocytes and the presence of a heterochromatic W-body in female somatic interphase nuclei, which are absent in males. The chromosomes are indistinguishable in different stages of both mitotic and meiotic divisions, except for orcein stained pachytene, where different bivalents can be differentiated to a certain degree. We regularly identified two distinctive bivalents that were associated with two different nucleoli in female pachytene spreads. One of these nucleoli is associated with the WZ bivalent and the other with an autosome bivalent. The autosome bivalent carried a terminally located NOR that was associated with small but clear heterochromatin. The presence of heterochromatin at the NORs is common in animals (e.g. [55], [56]) but in Lepidoptera has been reported only in the silkworm *B. mori* [57].

It remained unclear whether the sex-linked NOR of *B. anynana* was located on the W- or on the Z chromosome or on both sex chromosomes since we did not examine pachytene spermatocytes for a comparison. Due to the circular form of the WZ bivalent it was not possible to determine whether the sex-linked NOR is terminal or interstitial. Nevertheless, we favor location of the sex-linked NOR on the Z chromosome as the W chromosome appears composed entirely of heterochromatin, which would inhibit a high transcriptional activity of the active NOR.

The pachytene WZ bivalent of *B. anynana* is exceptional due to the tiny W chromosome. The W chromosome of the oriental tussock moth, *Artaxa subflava* is about half the size of the Z chromosome [58] and in the other lepidopteran species examined so far, the W chromosome was either only slightly smaller or comparable in size to the Z chromosome (e.g. [23], [59], [60]. Compatible lengths in the pachytene stage of such relatively similar sized W and Z chromosomes undoubtedly facilitate their complete pairing. A regular synaptonemal complex can be formed in spite of their obvious non-homology by means of twisting and synaptic adjustment [52], [61]. However, the size difference of W and Z is too large in *B. anynana* to form a regular bivalent. Instead, the much longer Z chromosome often forms a circle or horseshoe structure with the W chromosome closed inside. This arrangement could be considered an extreme case of synaptic adjustment as it allows the sex chromosomes to pair along their entire length. A similar mode of pairing was observed in mutants of the flour moth (*Ephestia kuehniella*), in which the W chromosome was shortened by irradiation [62], and also in *A. subflava*, in which the W chromosome comprises about half of the Z chromosome but shows still a conspicuous heterochromatic mass (see Fig. 3 in [58]). On the other hand, we cannot exclude that the W and Z chromosomes pair by means of some sequence homology, for example, in telomeric regions or via rDNA in the case of shared NORs. The *B. anynana* W chromosome is composed of constitutive heterochromatin as in many other Lepidoptera. This observation, combined with recent findings on the composition of W chromosomes in *B. mori*, *C. pomonella*, and several pyralids [57], [60], [63], [64], suggests that the *B. anynana* W chromosome is probably gene-poor and rich in interspersed repetitive sequences, such as transposable elements, which are known to be abundant in *B. anynana* in general [65]. The small size of the W chromosome is also reflected by a small heterochromatin body in Malpighian tubule nuclei of females. The size could indicate an intermediate stage in the process of secondary loss of the W chromosome as is the case in Lepidoptera that have adopted a ZZ/Z sex determination system after loss of the W chromosome [11].

### Linkage map

#### How to get the most out of an F_2_ design

The full-sib F_2_ cross design was chosen for the purpose of mapping QTL for ventral eyespot size. It generates a maximum phenotypic range in the offspring while keeping random genetic variation to a minimum. As a downside, this design is not ideally suited for linkage mapping with dominant markers.

One effect of having just one set of grandparents is that BI markers carry information in only one of both paternal linkage phases for most LGs (Supplement S8). Another effect is that it creates a strong bottleneck, that results in a lower proportion of FI and MI markers relative to BI markers than in an outbred cross (Supplement S8). This is most striking when the F_1_ male and female inherit the same set of P chromosomes, where 1∶1 segregating markers can only arise as a result of recombination in the P-male. This unfavorable F_1_ gamete combination occurs in 25% of the chromosomes, and is reflected by the complete absence of FI-markers in six LGs. Without recombination in the P-male for such LGs, generating more AFLP markers will not produce FI-markers because they do not exist for these linkage groups. Therefore, the chromosome prints for these six FI-devoid autosomes had to be obtained from BI and MI-markers instead. This reconstruction is based on the forbidden genotype restrictions, and the assumptions that either the unassigned individuals received a MI-marker that was fully associated with a non-recombinant BI-marker region (BI+MI reconstruction), or that the female BI component segregation is 1∶1 (BI only reconstruction). Empirical tests based on LGs with available chromosome prints showed that this approach creates chromosome prints that are identical or nearly identical to the available ones, and linkage maps that are very similar to those based on conventionally censored datasets. The stochastic deviations from the 1∶1 segregation have a negligible effect on the mapping distance and no effect on the mapping order. This validates the BI censoring approach for LGs without FI-markers.

The selective genotyping approach was chosen to avoid genotyping intermediate eyespot phenotypes in the offspring, since they provide hardly any additional information in QTL mapping compared to that of the extreme phenotypes [66]. As a result of this, the linkage map itself is based on a non-random set of offspring. The effect of this on the reliability of the linkage map is negligible because it does not affect the three main characteristics in linkage mapping: namely, marker grouping, marker order and marker distance. There could, however be an effect of selection on the ratios of segregating markers, since dominance promotes extreme phenotypes in recessive homozygotes and additive alleles produce extreme phenotypes in both types of homozygotes. Markers that are linked to genes which are involved with eyespot formation may therefore deviate from 3∶1 or 1∶1 ratios due to hitch-hiking.

#### Effects of data censoring

Using MAPMAKER with censored BI-markers as missing data resulted in a map that was 38% larger in size than the one produced from two subsets per chromosome with JOINMAP. This size difference is caused by two factors. Firstly, there is a software effect (i.e. algorithms used) that is revealed by analyzing only the (uncensored) MI-markers, that accounts for 17% of the difference in this study. The rest of the difference is caused by the treatment of the incompatible bi-phasic censored BI-markers. The main purpose of the MAPMAKER analysis was to allow comparison of mapping distance in *B. anynana* with other Lepidoptera linkage maps, since this is the first species in this taxon for which the final mapping step was performed in JOINMAP. This software has not been used before for Lepidoptera linkage maps, presumably because it is less able to deal with a substantial portion of non-overlapping genotypes than MAPMAKER. Our approach avoided this problem by adapting that of [34] which involves splitting up the dataset based on chromosome print value and omitting female-derived information rather than treating it as missing data. This results in two linkage maps per chromosome that are then juxtaposed and integrated based on common MI and codominant markers and their average distances. Rather than just using the average distance between the anchoring markers to combine the two phases, JOINMAP also takes the number of individuals representing both subsets into account [67].

#### Linkage groups and chromosome number

The number of LGs matches the karyotype, thus markers are available for all 27 autosomes and the Z chromosome. There are no markers available for the W chromosome, probably due to its small size. The marker densities and distances vary substantially between the different chromosomes, but given the uniform lengths of the pachytene bivalents, we interpret this as incomplete marker coverage rather than a difference in chromosome size. We aimed to present an integrated linkage map, with relative marker positions and distances based on both sets of incompatible BI-markers linked together with MI, codominant AFLP and microsatellite markers. We succeeded for 20 LGs, and mapped the remaining eight separately because they lacked sufficient anchoring markers. The presence of the *Tpi* gene of *B. anynana* is consistent with all (distantly related) Lepidoptera species for which this gene has been mapped to date (summarized in [11]). This strengthens the hypothesis of taxon-wide conserved synteny for at least part of the Lepidoptera Z chromosome.

#### Linkage and physical maps in Lepidoptera

The present linkage map provides the basis for the assignment of the number, position, effect and interactions of QTLs involved with the development of wingspot size. We will further anchor the map using SNP markers [68], with a main focus on genes that are involved in eyespot formation in *B. anynana* and eyespot and wing pattern formation in Lepidoptera in general. Additionally, physical anchoring of linkage groups to specific chromosomes by means of BAC-FISH, as has been performed in *B. mori* [35], will provide a solid framework for future mapping studies.

The MAPMAKER mapping distance of 1873 cM in *B. anynana* is within the 1430–2542 cM range reported for other butterfly species [25], [26], [37]. The accuracy of these mapping distances may however be limited, since mapping distances of both 1430 cM and 2400 cM were reported in *Heliconius erato* [26], [36] and distances ranging from 1305 cM to 6512 cM in *Bombyx mori* [30], [32] when using MAPMAKER software. One mapping software package that does support sex-specific map construction is CRI-MAP [69], which has been used to build many mammalian genetic maps. To our knowledge, CRI-MAP has never been used to compute a Lepidoptera map based on dominant markers. CRI-MAP shares some of its origins with MAPMAKER and suffers from the same deficiencies of MAPMAKER we have explained above. Notably, CRI-MAP includes (1) no robust method to choose an initial order of markers and (2) no systematic method to decide whether a marker should be excluded from the map because it cannot be reliably ordered. Our proposed mapping strategy avoids Lepidoptera specific issues that have an effect on mapping distance and order, but it still requires a large number of analysis steps. Therefore, we would welcome the implementation of sex-specific recombination in the analysis parameters of JOINMAP. This would not just be an asset to linkage mapping in Lepidoptera, but for all organisms in which sex-specific recombination rates have been reported.

## Supporting Information

Supplement S1 Haploid chromosome numbers of different Bicyclus species and their geographical origin.
(0.04 MB DOC)

Supplement S2 Reconstruction of the chromosome print
(0.05 MB DOC)

Supplement S3 Forbidden genotype screening
(0.05 MB DOC)

Supplement S4 Censoring of BI markers and map integration with anchoring markers
(0.12 MB DOC)

Supplement S5 Microsatellite censoring
(0.02 MB DOC)

Supplement S6 Implications of sliding window analysis of a “missing data”-censored dataset based on an example.
(0.24 MB DOC)

Supplement S7 Linkage group sizes of Bicyclus anynana produced with MAPMAKER and with JOINMAP
(0.09 MB DOC)

Supplement S8 Implications of a full-sib design
(0.08 MB DOC)
