# Supplementary material for: Cytogenetic Characterization and AFLP-Based Genetic Linkage Mapping for the Butterfly Bicyclus anynana, Covering All 28 Karyotyped Chromosomes
Source: PLoS One. 2008 Dec 8;3(12):e3882. doi: 10.1371/journal.pone.0003882 (PMC2588656; doi:10.1371/journal.pone.0003882)
Supplement: Supplement S1 — Haploid chromosome numbers of different Bicyclus species and their geographical origin. (0.04 MB DOC) [file pone.0003882.s001.doc]

**Supplement 1. Haploid chromosome numbers of different Bicyclus species and their geographical origin**

| species | n | Origin | Reference |
| --- | --- | --- | --- |
| *B. auricrudus parvocellata* | *ca* 14[[1]](#footnote-2) | Entebbe (Uganda) | De Lesse 1968 |
| *B. jefferyi* | 28 | Entebbe (Uganda) | De Lesse 1968 |
| *B. sophrosyne* | 28 | Entebbe (Uganda) | De Lesse 1968 |
| *B. mollita* | 28 | Entebbe (Uganda) | De Lesse 1968 |
| *B. safitza* | 28 | Entebbe (Uganda) | De Lesse 1968 |
| *B. dentatus* | 29 | Fort Portal (Uganda);  Katamayo Forest (Kenya) | De Lesse 1968 |
| *B. anynana* | 28 | Entebbe (Uganda) | De Lesse 1968 |
| *B. funebris agraphis* | 28 | Entebbe (Uganda) | De Lesse 1968 |
| *B. funebris funebris* | 29 | Forêt de Tobor (Senegal) | De Lesse and Condamin 1962 |
| *B. saussurei* | 28 | Entebbe (Uganda) | De Lesse 1968 |
| *B. zinebi* | 26 | Forêt de Santiaba-Mandjak (Senegal) | De Lesse and Condamin 1965 |
| *B. vulgaris* | 28 | Lamto (Ivory Coast);  Forêt de Tobor (Senegal) | De Lesse and Condamin 1965  De Lesse and Condamin 1962 |
| *B. sandace* | 28 | Sangalkam (Senegal) | De Lesse and Condamin 1962 |

1. De Lesse 1968: “Quant à *B. auricrudus*, à n = *ca* 14, il représente sans doute, comme ailleurs (cf. *Eurema brigitta*, p 592), un cas de réunion de chromosomes.” This translates to: As for *B. auricrudus*, with n = *ca* 14, it represents without a doubt, as in others (cf. *Eurema brigitta*, p 592), a case of chromosome fusion. [↑](#footnote-ref-2)
