# Supplementary material for: Cytogenetic Characterization and AFLP-Based Genetic Linkage Mapping for the Butterfly Bicyclus anynana, Covering All 28 Karyotyped Chromosomes
Source: PLoS One. 2008 Dec 8;3(12):e3882. doi: 10.1371/journal.pone.0003882 (PMC2588656; doi:10.1371/journal.pone.0003882)
Supplement: Supplement S2 — Reconstruction of the chromosome print (0.05 MB DOC) [file pone.0003882.s002.doc]

**Supplement 2a. Reconstruction of the chromosome print based on forbidden genotype restrictions**

As a result of the full-sib cross design, one out of four linkage groups has a very small proportion of 1:1 informative markers, which makes it difficult to generate chromosome prints for these linkage groups. Without a chromosome print available, BI markers can still be mapped, but without censoring, the software will underestimate the mapping distance about twofold because absence of recombination from the female-derived genotypes is interpreted as close linkage. Therefore, the markers on these chromosomes need to be censored, but in an alternative way from the FI marker based censoring.


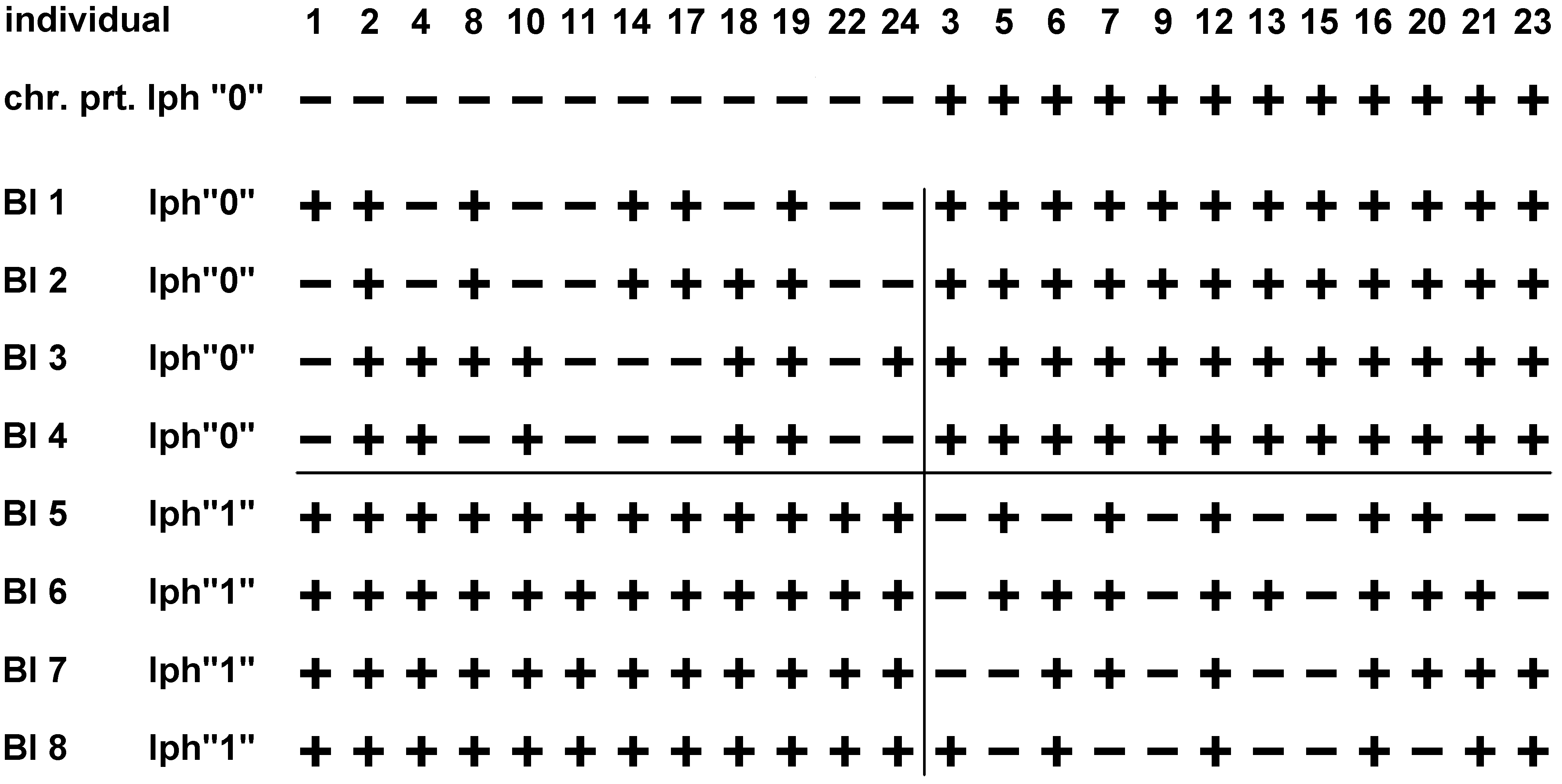


Fig. 4. Example of the relation between chromosome print and dominant marker phenotype of BI markers in the two different maternal linkage phases. Chromosome print values and the peakscores of eight BI markers of 24 F_2_ individuals (+ = peakpresent, – = peakabsent) are shown. All markers (and the chromosome print) belong to the same linkage group and both maternal linkage phases (lph“0” and lph“1”) are represented by the BI markers (four of each). All markers have the same paternal linkage phase. The individuals have been grouped based on their chromosome print value and maternal linkage phase. The grouping reveals two clusters with exclusively peakpresents and two clusters with a mixture of peakpresents and peakabsents. This figure shows the pattern on which the reconstruction is based and not the actual reconstruction itself. Thus individuals 2, 12, 16 and 19 stay unassigned when using this pattern for reconstruction without chromosome prints and without additional (e.g. MI) information.

Given the forbidden genotype restrictions, an individual that has one or more peakabsents in one maternal linkage phase of BI markers must have exclusively peakpresents in the alternative maternal linkage phase. This peakabsent-linkage phase combination gives a pattern from which a substantial part of the chromosome print can be reconstructed. This pattern is shown in Fig. 4 where markers with the same linkage phases are grouped vertically and the individuals with the same chromosome print values horizontally. The pattern is a direct consequence of the absence of recombination in the females. A “–” chromosome print value combined with BI marker in linkage phase “1” produces exclusively peakpresents (bottom left). A chromosome print “+” together with BI linkage phase “0” also gives exclusively peakpresents (top right). These two “exclusively peakpresent” clusters consist of a mixture of heterozygotes and homozygote peakpresents that cannot be told apart. The remaining genotypes (top left and bottom right) are either homozygous peakabsent or heterozygotes that inherited a paternal peakpresent (and a maternal peakabsent). The chromosome print values can be reconstructed directly from this pattern for individuals that have at least one peakabsent and known linkage phases. The remaining individuals have peakpresent values for all markers in both maternal linkage phases (e.g. individuals 2, 12, 16 and 19 in Fig. 4), so that distinction between F_2_ male- and female component is not possible and the chromosome print values for these individuals stay “unassigned”. The proportion of individuals with “all-present” in both linkage phases is substantial in a full-sib cross as explained in Supplement 8. They reflect non-recombinant regions on the F_1_ male chromosome. In contrast, all-presents are far less abundant in outbred crosses, which makes the reconstruction of a chromosome print easier, but it will generally not be necessary to reconstruct them in the first place because FI markers are more common in outbred crosses.

**2b. Reconstruction of the chromosome print for unassigned individuals with only BI markers available**

The unasigned individuals are all-peakpresent in both linkage phases of BI markers. Therefore, it remains unclear what linkage phase should be censored. However, it does not have any consequences for the linkage map whether the actual female component is censored, or whether an equal number of random “all-peakpresents” is excluded. This means that for half these unassigned individuals, the chromosome print can be set to peakabsent and for the other half to peakpresent, based on the assumption that the female marker inheritance is 1:1. In case of the example given in Fig. 4, individuals 2 and 12 would be assigned chromosome print value “–” and 16 and 19 assigned value “+”. This means that 12 and 19 are grouped in the wrong cluster, but this has no consequences for marker distribution, and therefore no effect on the linkage map. In addition, because stochastic departures from the 1:1 ratio have an effect on the mapping distance, chromosome prints were reconstructed based on the extremes of the binomial 95% confidence interval for 1:1 segregation in order to determine the error margins of this approach. Shifting the censoring to the boundaries of the 95% binomial confidence level changed the total mapping distance 2 cM or less, and kept the marker order intact. We applied the same reconstruction method to the first ten linkage groups (with chromosome prints available) to determine the consistency of mapping order and distance for the BI-only linkage groups when using this technique.

**2c. Reconstruction of the chromosome print for unassigned individuals with BI and MI markers available**

The random 1:1 reconstruction described in Supplement 2b is only suitable for linkage groups with exclusively BI markers. It is not appropriate for a combination of BI and MI markers, because there is an effect on the linkage map when MI markers end up in the wrong censoring group due to random chromosome print value assignment. However, with MI markers available, the chromosome print values of the unassigned individuals can also be determined. For the following reconstruction, we take only BI markers with one of the two possible paternal linkage phases into account. We choose the paternal phase with the most representatives available, but with a full-sib design, this usually means that all markers are included because the full-sib cross causes most BI markers to be in the same paternal phase (Supplement 8). Specifically, this means that we only look at markers that are peakpresent on the same F_1_ male chromosome and leave out the markers that are peakpresent on the other. An individual that inherited BI markers that are all in a non-recombinant region of the F_1_ male chromosome will be either exclusively peakpresent or exclusively peakabsent within a maternal linkage phase. In Fig. 4, the non-recombinant “all peakpresents” are represented by individuals 2, 12, 16 and 19 and the non-recombinants with exclusively peakabsents in one maternal phase are represented by individuals 9, 11, 15 and 22. A MI marker that lies within such a homogenous (non-recombinant) region will share the same pattern, with exclusively MI peakpresents associated with BI all-peakabsents in one phase, and MI peakabsents with BI all-peakabsents in the opposite phase. Thus by using the pattern of MI loci that meet these criteria, the male and female component in the unassigned (BI all-present) individuals can be determined, which allows reconstruction of the chromosome print for all unassigned individuals. This approach to identify the male component of F_2_ markers is similar to the identification of the female component based on FI markers (and chromosome prints). The main difference is that FI markers are always fully linked to BI markers because all female inherited chromosomes are completely non-recombinant while identification of the male component is based on non-recombinant chromosome regions that need to be identified first.

In order to test and validate the BI+MI-based chromosome print reconstruction empirically, we used the first ten linkage groups (with chromosome prints available) to determine the difference between the actual chromosome print and reconstructed chromosome print for BI+MI linkage groups.
