# Supplementary material for: Cytogenetic Characterization and AFLP-Based Genetic Linkage Mapping for the Butterfly Bicyclus anynana, Covering All 28 Karyotyped Chromosomes
Source: PLoS One. 2008 Dec 8;3(12):e3882. doi: 10.1371/journal.pone.0003882 (PMC2588656; doi:10.1371/journal.pone.0003882)
Supplement: Supplement S3 — Forbidden genotype screening (0.05 MB DOC) [file pone.0003882.s003.doc]

**Supplement 3. Forbidden genotype screening**

The manifestation of a forbidden genotype combination of a BI marker and the chromosome print depends on their relative linkage phases in the F_1_ female. When the chromosome print positive signal and the BI marker positive signal are on the alternate chromosomes in the F_1_ female, the offspring must always be positive peakpresent either in the chromosome print, screened marker, or in both. Both absent would mean that the marker negative signals formed a novel combination (i.e. both on the same chromosome) in the female, and thus resulted from forbidden recombination (Fig. 5a). Similarly, both


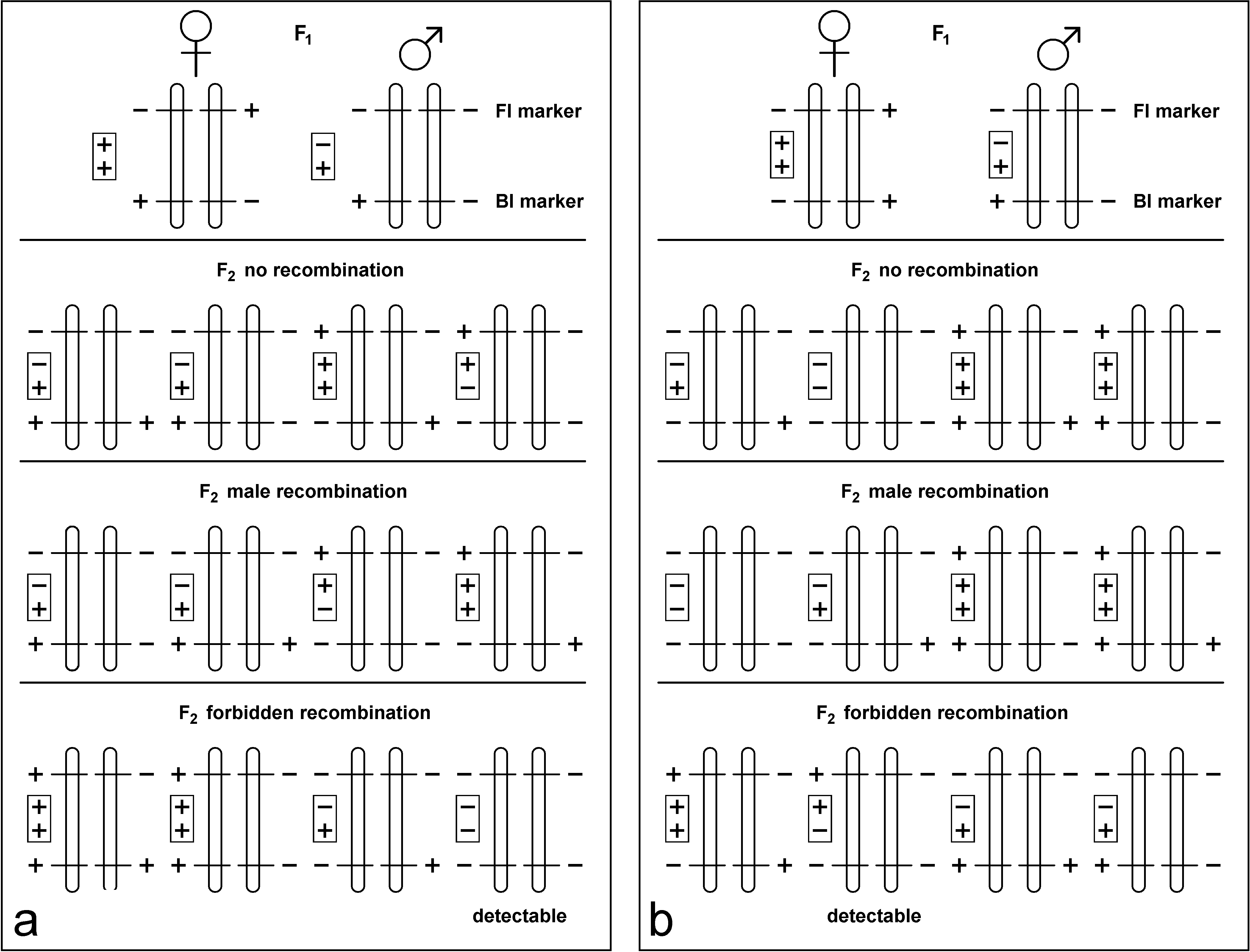


Fig 5. Detection of forbidden genotypes. (a) Cross between an F_1_ female with positive signals in repulsion and an F_1_ male with one positive BI signal. Vertical bars represent chromosomes with marker loci on both ends. The chromosome print is here represented by an FI marker. The characters in the box represent the dominant marker output (i.e. + = peakpresent, – = peakabsent) for both loci. Male recombination does not result in new marker combinations and is only included for completeness. Of the four possibilities, only the double negative marker combination is distinguishable as forbidden genotype; (b) Cross with the positive signals linked in the F_1_ female. In this case, an FI peakpresent combined with a BI peakabsent is detectable as a forbidden recombination.

positive signals on the same chromosome would also indicate forbidden recombination. However, such a haplotype does not give a unique detectable combination with dominant markers. In fact, only one out of four forbidden genotype combinations can be detected when using dominant markers (Fig. 5a). The alternative F_1_ female marker combination has the positive signals on the same chromosome, and both negatives on the other (Fig. 5b). In this case, absence of the BI marker in combination with presence of the chromosome print marker is not allowed. Again, only one out of four forbidden genotypes is detected. Therefore we excluded loci with more than two inconsistencies from further analysis. Forbidden genotype screening in microsatellites is similar, but the proportion of detectable forbidden genotypes is higher (50% or 100% depending on the number of alleles involved).
