# Supplementary material for: Cytogenetic Characterization and AFLP-Based Genetic Linkage Mapping for the Butterfly Bicyclus anynana, Covering All 28 Karyotyped Chromosomes
Source: PLoS One. 2008 Dec 8;3(12):e3882. doi: 10.1371/journal.pone.0003882 (PMC2588656; doi:10.1371/journal.pone.0003882)
Supplement: Supplement S4 — Censoring of BI markers and map integration with anchoring markers (0.12 MB DOC) [file pone.0003882.s004.doc]

**Supplement 4. Censoring of BI markers and map integration with anchoring markers**

BI markers that have F_1_ female inherited peakpresents must be excluded from analysis because the absence of recombination in females makes them uninformative. The chromosome print defines which individual-linkage phase combinations have such an allele. The pattern shown in Fig. 4 that was used for chromosome print reconstruction, also groups


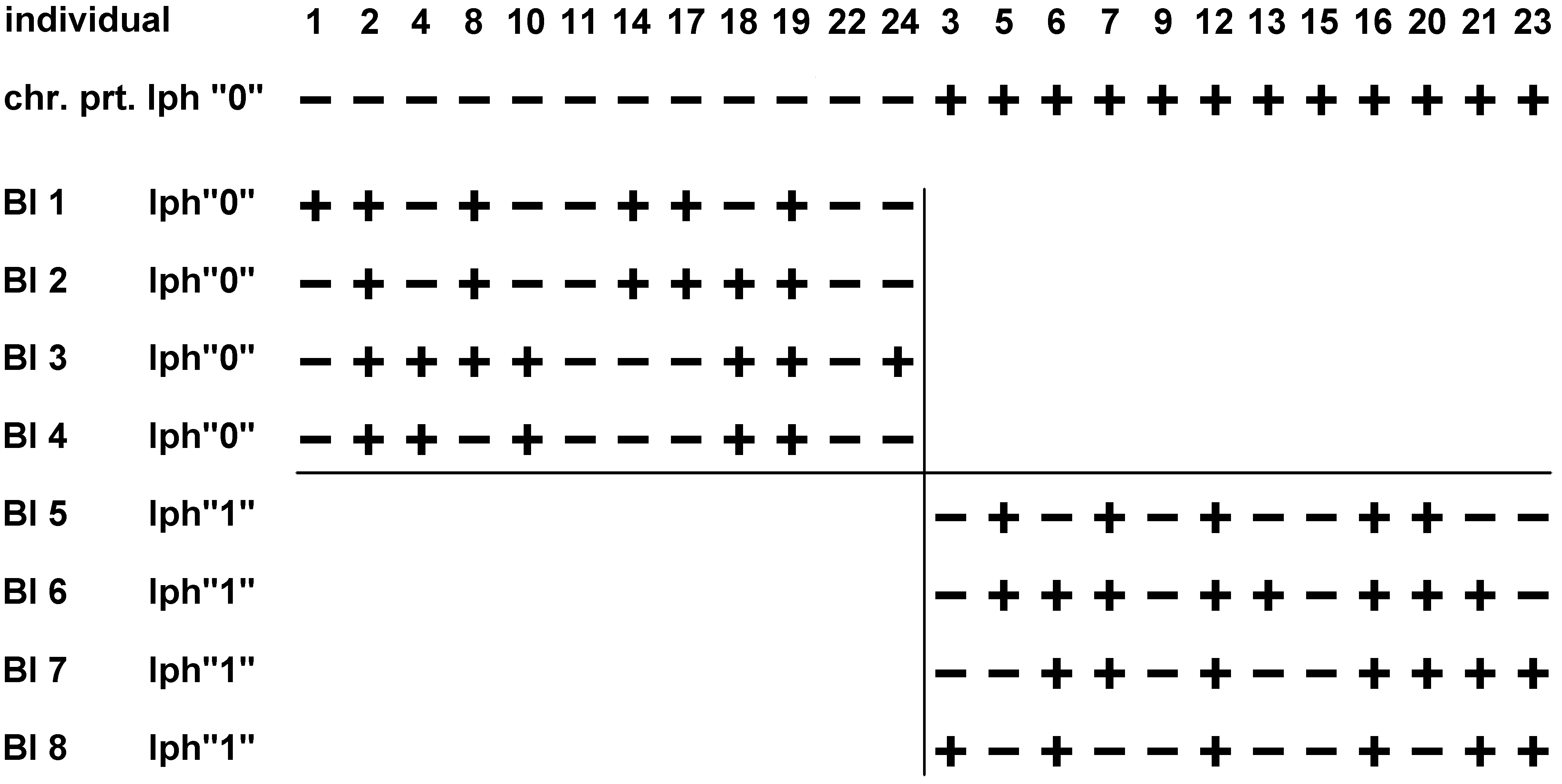


Fig. 6. Censored BI markers. The same diagram as in Fig. 4, but now with the uninformative clusters removed.

the data that is to be excluded from analysis. The “exclusively peakpresent” clusters with a female inherited component that need to be censored are shown in Fig. 6. The issue of two incompatible linkage groups per chromosome is also clearly illustrated in Fig. 6. Excluding the two peakpresent clusters from analysis leaves two subsets of data (top left and bottom right) that cannot be linked to each other without anchoring markers because they do not hold information within the same individuals. Fig. 7 shows two MI markers in addition to the eight BI markers in Fig. 6. They provide the information that allows integration of the two


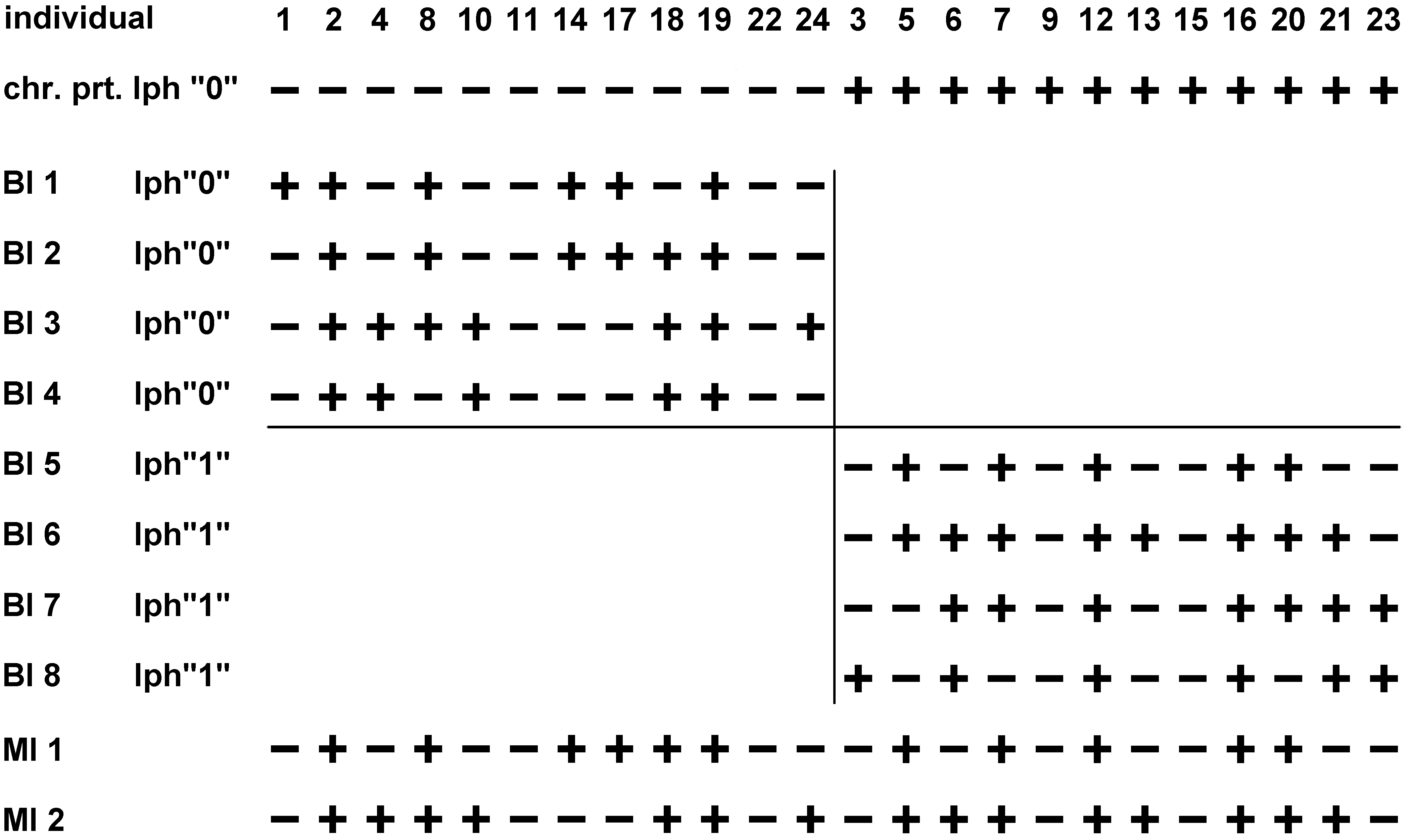


Fig. 7. Censored BI markers with MI anchoring markers. With anchoring markers (here represented by MI markers), both BI clusters can be linked together and integrated into a single linkage group because the MI markers are informative in all individuals. The linkage phase of the MI markers is not shown here because MI markers do not have a maternal linkage phase and the paternal linkage phase is not relevant in this example.

clusters because their genotypes are informative in all F_2_ individuals. In our approach, we first construct two separate linkage maps. One for individuals with chromosome print value “–” and the other for individuals with chromosome print value “+”.With at least two anchoring markers that are not closely linked available, they can be integrated by JOINMAP as demonstrated in Fig. 8.


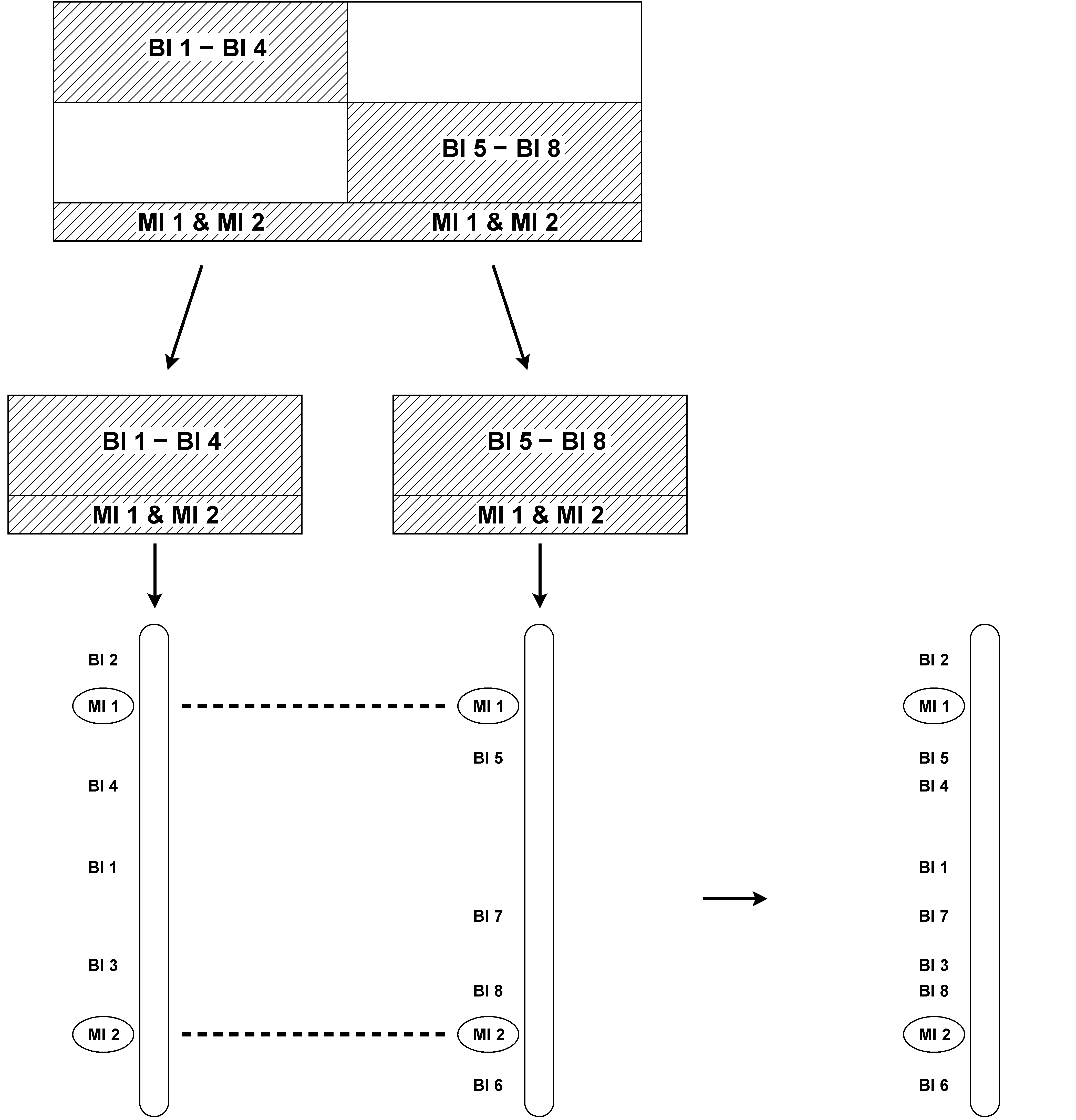


Fig 8. Mapping and Integration of two incompatible BI linkage groups. This is a schematic representation of the censored data as shown in Fig. 7, followed by the final mapping steps. Individuals with different chromosome print values are mapped separately. The two anchoring (MI) markers facilitate integration into a single linkage map.
