# Supplementary material for: Cytogenetic Characterization and AFLP-Based Genetic Linkage Mapping for the Butterfly Bicyclus anynana, Covering All 28 Karyotyped Chromosomes
Source: PLoS One. 2008 Dec 8;3(12):e3882. doi: 10.1371/journal.pone.0003882 (PMC2588656; doi:10.1371/journal.pone.0003882)
Supplement: Supplement S5 — Microsatellite censoring (0.02 MB DOC) [file pone.0003882.s005.doc]

**Supplement 5. Microsatellite censoring**

Microsatellite genotypes need to be converted so that only the paternal component in the F_2_ is used for mapping. F_1_-male-specific alleles can be translated directly into a present-absent pattern for that allele, and then used as a MI marker.

When the F_1_ male and female are both heterozygous with the same sets of alleles, it is not immediately clear which allele came from which parent in the F_2_ heterozygotes. However, the chromosome print can reveal the origin of these heterozygous alleles. The two alternative F_2_ heterozygotes are fully associated with the opposite chromosome print values. Distinction between the male and female component is illustrated by an example below.

We consider a microsatellite locus with a 10 repeat allele and a 12 repeat allele in both F_1_ male and F_1_ female.

F_1_ female 10^a^/12^a^

F_1_ male 10^b^/12^b^

F_2_ (offspring) 10^a^/10^b^ 10^a^/12^b^ 12^a^/10^b^ 12^a^/12^b^

Chromosome print – – + +

Translated to MI: 10 12 10 12

The 10/10 F_2_ individuals should all have the same chromosome print values (e.g. “–”) and the 12/12 F_2_ individuals should all have the opposite chromosome print values (i.e. “+”). This homozygote-chromosome print combination reveals which chromosome print value is associated with the maternal “10^a^” allele and which with the maternal “12^a^” allele. The chromosome print-allele association is the same in the F_2_ heterozygotes and thus reveals the female component that is to be excluded from analysis. This codominant censoring does not result in the exclusion of half the individuals for such a locus, as is the case in BI marker censoring. Instead, these microsatellites can be treated as MI markers representing all F_2_ individuals, and serve as anchoring markers.
