# Supplementary material for: Cytogenetic Characterization and AFLP-Based Genetic Linkage Mapping for the Butterfly Bicyclus anynana, Covering All 28 Karyotyped Chromosomes
Source: PLoS One. 2008 Dec 8;3(12):e3882. doi: 10.1371/journal.pone.0003882 (PMC2588656; doi:10.1371/journal.pone.0003882)
Supplement: Supplement S6 — Implications of sliding window analysis of a “missing data”-censored dataset based on an example. (0.24 MB DOC) [file pone.0003882.s006.doc]

**Supplement 6. Implications of sliding window analysis of a “missing data”-censored dataset based on an example.**

Linkage group 21 is used to illustrate the consequences of sliding window analysis on a linkage group that has no evenly spaced anchoring markers. Fig. 9 shows the linkage map of LG21 with anchoring markers and BI markers of both phases in different colors. A sliding window of five neighboring markers usually results in incompatible combinations. Table 2 shows the censored genotype score of all LG21 markers in 14 F_2_ individuals. Below that are three examples of marker subsets that are compared with different sliding window positions (Table 3a-3c). The first two examples allow reliable marker ordering, but the third example shows why a missing data approach can have severe consequences for the reliability of the marker order. The mapping approach described in Supplement 4 avoids these compatibility issues because it only compares markers that have genotype scores available within the same individuals.


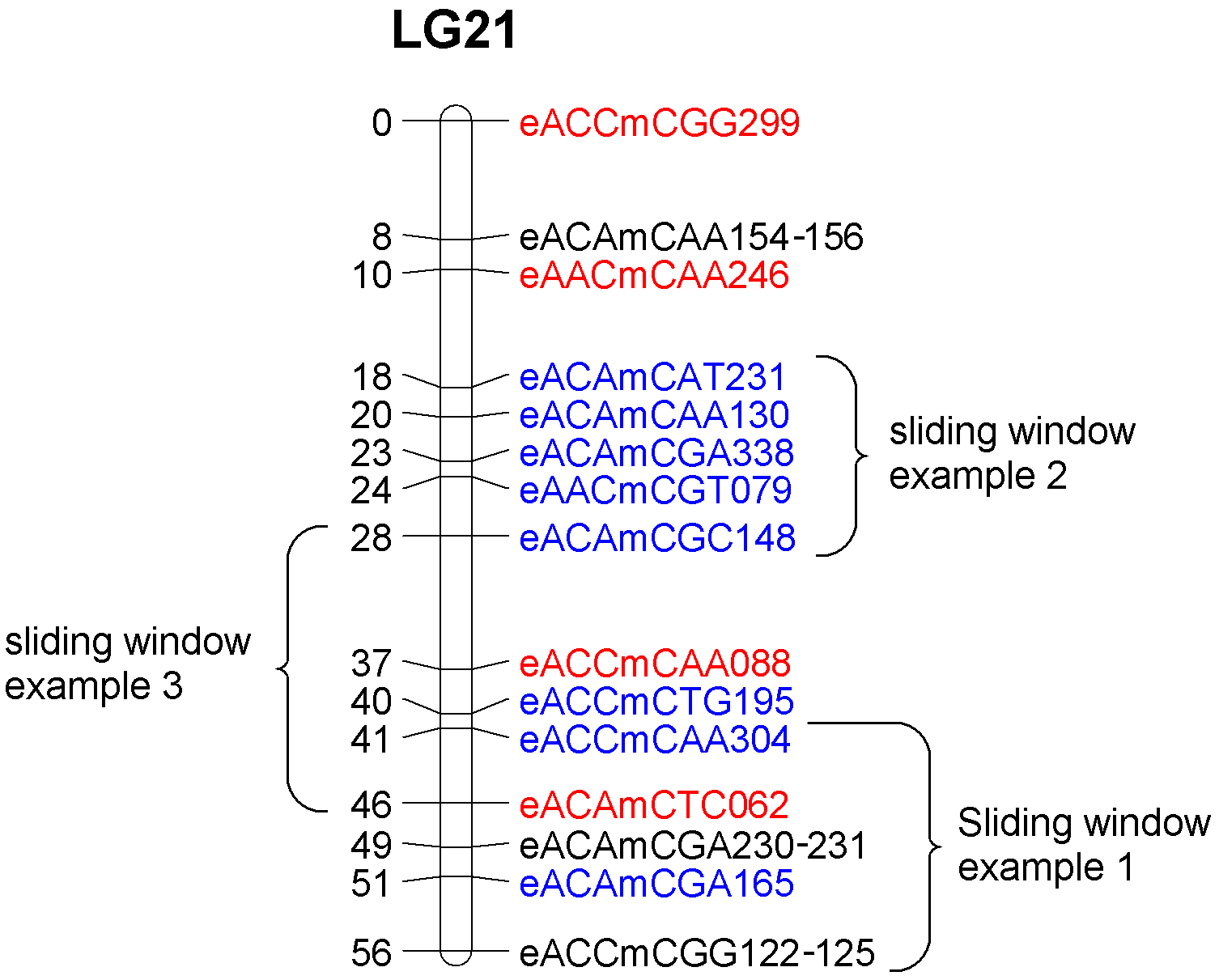


Fig. 9. Mapping order, mapping distance and maternal linkage phase of BI markers in linkage group 21. Mapping order and distance are based on separate phase mapping followed by integration in JOINMAP. Markers in red have maternal linkage phase “0”, markers in blue have maternal linkage phase “1”. Markers in black are codominant. The linkage phase of the codominant markers is not relevant in this context. The three subsets of five markers that are dealt with in the sliding window examples below are indicated with braces.

Table 2. Censored peakscore of LG21.

The marker order and color scheme of the linkage phases is the same as in Fig. 9, with red and blue representing opposite maternal linkage phases. The censored values are highlighted in purple. The codominant markers have been merged to show only their male informative component. They have a genotype score in all individuals. Only 14 of the 92 individuals are shown. Missing data (md) in the markers in red corresponds with chromosome print value “0” and in the blue markers with chromosome print value “1”.

| **F_2_ Individual** | 1 | 2 | 3 | 4 | 5 | 6 | 7 | 8 | 9 | 10 | 11 | 12 | 13 | 14 |
| --- | --- | --- | --- | --- | --- | --- | --- | --- | --- | --- | --- | --- | --- | --- |
|  | | | | | | | | | | | | | | |
| **eACCmCGG299** | md | md | md | 0 | 0 | md | 0 | md | md | md | 1 | 0 | md | 0 |
| **eACAmCAA154-156** | 0 | 1 | 0 | 0 | 0 | 1 | 1 | 0 | 1 | 0 | 1 | 0 | 1 | 1 |
| **eAACmCAA246** | md | md | md | 0 | 0 | md | 0 | md | md | md | 1 | 0 | md | 1 |
| **eACAmCAT231** | 1 | 0 | 1 | md | md | 0 | md | 1 | 0 | 0 | md | md | 1 | md |
| **eACAmCAA130** | 1 | 0 | 1 | md | md | 0 | md | 0 | 0 | 0 | md | md | 1 | md |
| **eACAmCGA338** | 1 | 0 | 1 | md | md | 0 | md | 1 | 0 | 0 | md | md | 1 | md |
| **eAACmCGT079** | 1 | 1 | 1 | md | md | 0 | md | 1 | 0 | 0 | md | md | 0 | md |
| **eACAmCGC148** | 1 | 0 | 1 | md | md | 0 | md | 1 | 1 | 0 | md | md | 1 | md |
| **eACCmCAA088** | md | md | md | 0 | 0 | md | 1 | md | md | md | 1 | 0 | md | 1 |
| **eACCmCTG195** | 0 | 0 | 1 | md | md | 1 | md | 1 | 0 | 0 | md | md | 1 | md |
| **eACCmCAA304** | 1 | 1 | 1 | md | md | 0 | md | 1 | 0 | 1 | md | md | 0 | md |
| **eACAmCTC062** | md | md | md | 0 | 0 | md | 1 | md | md | md | 1 | 0 | md | 1 |
| **eACAmCGA230-231** | 0 | 0 | 1 | 0 | 0 | 1 | 1 | 0 | 1 | 0 | 1 | 0 | 1 | 1 |
| **eACAmCGA165** | 1 | 1 | 0 | md | md | 0 | md | 1 | 0 | 1 | md | md | 0 | md |
| **eACCmCGG122-125** | 0 | 0 | 1 | 0 | 0 | 1 | 1 | 0 | 1 | 0 | 1 | 0 | 1 | 0 |
|  | | | | | | | | | | | | | | |
| Chromosome print 21 | 0 | 0 | 0 | 1 | 1 | 0 | 1 | 0 | 0 | 0 | 1 | 1 | 0 | 1 |

Table 3a. Sliding window example 1

These are the five markers at the bottom of the linkage group. This includes two anchoring markers, two BI markers with maternal linkage phase “1” and one with maternal linkage phase “0”. The mapping order can be determined for all markers due to the presence of two anchoring markers.

| **F_2_ Individual** | 1 | 2 | 3 | 4 | 5 | 6 | 7 | 8 | 9 | 10 | 11 | 12 | 13 | 14 |
| --- | --- | --- | --- | --- | --- | --- | --- | --- | --- | --- | --- | --- | --- | --- |
|  | | | | | | | | | | | | | | |
| **eACCmCAA304** | 1 | 1 | 1 | md | md | 0 | md | 1 | 0 | 1 | md | md | 0 | md |
| **eACAmCTC062** | md | md | md | 0 | 0 | md | 1 | md | md | md | 1 | 0 | md | 1 |
| **eACAmCGA230-231** | 0 | 0 | 1 | 0 | 0 | 1 | 1 | 0 | 1 | 0 | 1 | 0 | 1 | 1 |
| **eACAmCGA165** | 1 | 1 | 0 | md | md | 0 | md | 1 | 0 | 1 | md | md | 0 | md |
| **eACCmCGG122-125** | 0 | 0 | 1 | 0 | 0 | 1 | 1 | 0 | 1 | 0 | 1 | 0 | 1 | 0 |

Table 3b. Sliding window example 2

Five neighboring BI markers with the same maternal linkage phase. Marker order and integrity can be determined for all five markers in this subset because the informative genotype scores are available for all individuals.

| **F_2_ Individual** | 1 | 2 | 3 | 4 | 5 | 6 | 7 | 8 | 9 | 10 | 11 | 12 | 13 | 14 |
| --- | --- | --- | --- | --- | --- | --- | --- | --- | --- | --- | --- | --- | --- | --- |
|  | | | | | | | | | | | | | | |
| **eACAmCAT231** | 1 | 0 | 1 | md | md | 0 | md | 1 | 0 | 0 | md | md | 1 | md |
| **eACAmCAA130** | 1 | 0 | 1 | md | md | 0 | md | 0 | 0 | 0 | md | md | 1 | md |
| **eACAmCGA338** | 1 | 0 | 1 | md | md | 0 | md | 1 | 0 | 0 | md | md | 1 | md |
| **eAACmCGT079** | 1 | 1 | 1 | md | md | 0 | md | 1 | 0 | 0 | md | md | 0 | md |
| **eACAmCGC148** | 1 | 0 | 1 | md | md | 0 | md | 1 | 1 | 0 | md | md | 1 | md |

Table 3c. Sliding window example 3

This subset contains markers of both maternal linkage phases but no anchoring markers. It is not possible to establish marker order or marker order integrity for all five markers because genotypes in markers of maternal phase “1” are always compared with missing data in markers with linkage phase “0” and vice versa. In such a case, JOINMAP refuses to suggest a marker order, but MAPMAKER will give a marker order even though it may not correspond with the actual order of the markers.

| **F_2_ Individual** | 1 | 2 | 3 | 4 | 5 | 6 | 7 | 8 | 9 | 10 | 11 | 12 | 13 | 14 |
| --- | --- | --- | --- | --- | --- | --- | --- | --- | --- | --- | --- | --- | --- | --- |
|  | | | | | | | | | | | | | | |
| **eACAmCGC148** | 1 | 0 | 1 | md | md | 0 | md | 1 | 1 | 0 | md | md | 1 | md |
| **eACCmCAA088** | md | md | md | 0 | 0 | md | 1 | md | md | md | 1 | 0 | md | 1 |
| **eACCmCTG195** | 0 | 0 | 1 | md | md | 1 | md | 1 | 0 | 0 | md | md | 1 | md |
| **eACCmCAA304** | 1 | 1 | 1 | md | md | 0 | md | 1 | 0 | 1 | md | md | 0 | md |
| **eACAmCTC062** | md | md | md | 0 | 0 | md | 1 | md | md | md | 1 | 0 | md | 1 |
