# Supplementary material for: Cytogenetic Characterization and AFLP-Based Genetic Linkage Mapping for the Butterfly Bicyclus anynana, Covering All 28 Karyotyped Chromosomes
Source: PLoS One. 2008 Dec 8;3(12):e3882. doi: 10.1371/journal.pone.0003882 (PMC2588656; doi:10.1371/journal.pone.0003882)
Supplement: Supplement S7 — Linkage group sizes of Bicyclus anynana produced with MAPMAKER and with JOINMAP (0.09 MB DOC) [file pone.0003882.s007.doc]

**Supplement 7. Linkage group sizes of *Bicyclus anynana* produced with MAPMAKER and with JOINMAP**

Mapping with MAPMAKER instead of JOINMAP resulted in larger mapping distances. With two incompatible linkage groups per chromosome, only the larger one was mapped.

The “MM” “MM err. det.” and “JM” columns give the linkage group sizes based on all informative markers. The three columns on the right report the male-informative mapping distances. The “err. det.” columns specify the MAPMAKER output with error detection activated. The MI-based linkage groups are generally smaller size, partly because the MI data is more reliable, but mainly because they are physically closer to each other than the BI markers at the extremes of the linkage groups.

|  | MM | MM  err. det. | JM | MM MI | MM MI  err. det. | JM MI |
| --- | --- | --- | --- | --- | --- | --- |
| LG01 | 138.8 cM | 97.1 | 71 cM | 88.5 cM | 66.1 cM | 54 cM |
| LG02 | 73.1 cM | 66.6 | 57 cM | 46.2 cM | 44.1 cM | 41 cM |
| LG03 | 95.2 cM | 77.6 | 68 cM |  |  |  |
| LG04 | 74.5 cM | 65.9 | 59 cM | 51.9 cM | 50.4 cM | 42 cM |
| LG05 | 78.7 cM | 74.5 | 65 cM |  |  |  |
| LG06 | 75.6 cM | 51.0 | 43 cM | 52.6 cM | 50.8 cM | 47 cM |
| LG07 | 74.8 cM | 60.9 | 48 cM | 63.9 cM | 60.8 cM | 51 cM |
| LG08 | 81.8 cM | 60 | 49 cM | 59.3 cM | 51.5 cM | 42 cM |
| LG09 | 99.3 cM | 77.6 | 70 cM | 71.3 cM | 64.6 cM | 52 cM |
| LG10 | 88.8 cM | 66.6 | 52 cM | 25.4 cM | 24.1 cM | 21 cM |
| LG11 | 18.0 cM | 14.3 | 14 cM |  |  |  |
| LG12 | 8.4 cM | 8.4 | 8 cM |  |  |  |
| LG13 | 104.2 cM | 102.4 | 84 cM | 31.8 cM | 31.8 cM | 29 cM |
| LG14 | 37.6 cM | 37.6 | 32 cM |  |  |  |
| LG15 | 63.4 cM | 46.7 | 38 cM | 11.0 cM | 11 cM | 10 cM |
| LG16 | 52.8 cM | 51.6 | 41 cM | 35.9 cM | 36.1 cM | 32 cM |
| LG17 | 73.5 cM | 68.6 | 35 cM |  |  |  |
| LG18 | 50.8 cM | 47.6 | 35 cM |  |  |  |
| LG19 | 82.7 cM | 94.5 | 57 cM | 12.3 cM | 12.3 cM | 11 cM |
| LG20 | 23.3 cM | 23.3 | 20 cM |  |  |  |
| LG21 | 108.5 cM | 83.9 | 56 cM |  |  |  |
| LG22 | 128.9 cM | 116.5 | 61 cM |  |  |  |
| LG23 | 62.6 cM | 54.8 | 43 cM |  |  |  |
| LG24 | 58.1 cM | 29.6 | 27 cM |  |  |  |
| LG25 | 111.5 cM | 122.2 | 57 cM | 31.0 cM | 31 cM | 25 cM |
| LG26 | 153.6 cM | 119.8 | 58 cM |  |  |  |
| LG27 | 107.0 cM | 86.1 | 53 cM | 15.1 cM | 15.1 cM | 13 cM |
| LGZ | 83.0 cM | 67.0 | 53 cM |  |  |  |
| Total | 2208.5 cM | 1872.7 | 1354 cM | 596.2 cM | 549.7 cM | 470 cM |
