# Supplementary material for: Cytogenetic Characterization and AFLP-Based Genetic Linkage Mapping for the Butterfly Bicyclus anynana, Covering All 28 Karyotyped Chromosomes
Source: PLoS One. 2008 Dec 8;3(12):e3882. doi: 10.1371/journal.pone.0003882 (PMC2588656; doi:10.1371/journal.pone.0003882)
Supplement: Supplement S8 — Implications of a full-sib design (0.08 MB DOC) [file pone.0003882.s008.doc]

**Supplement 8. Implications of a full-sib design**

The use of a full-sib design has implications for the availability and ratios of the different marker types (FI, MI, BI), and for the linkage phases of the informative markers. In an outbred full-sib design, there are four different (autosomal) chromosome sets present in the P-generation (Fig. 10). Consequently, there are sixteen possible non-recombinant chromosome combinations in the F_1_. Fig. 10 and 11 show only four of these combinations, because the other twelve (three sets of four) have the same implications. Each set contains: *i* F_1_ male and female with the same chromosome combination, *ii* F_1_ male and female with the same maternal


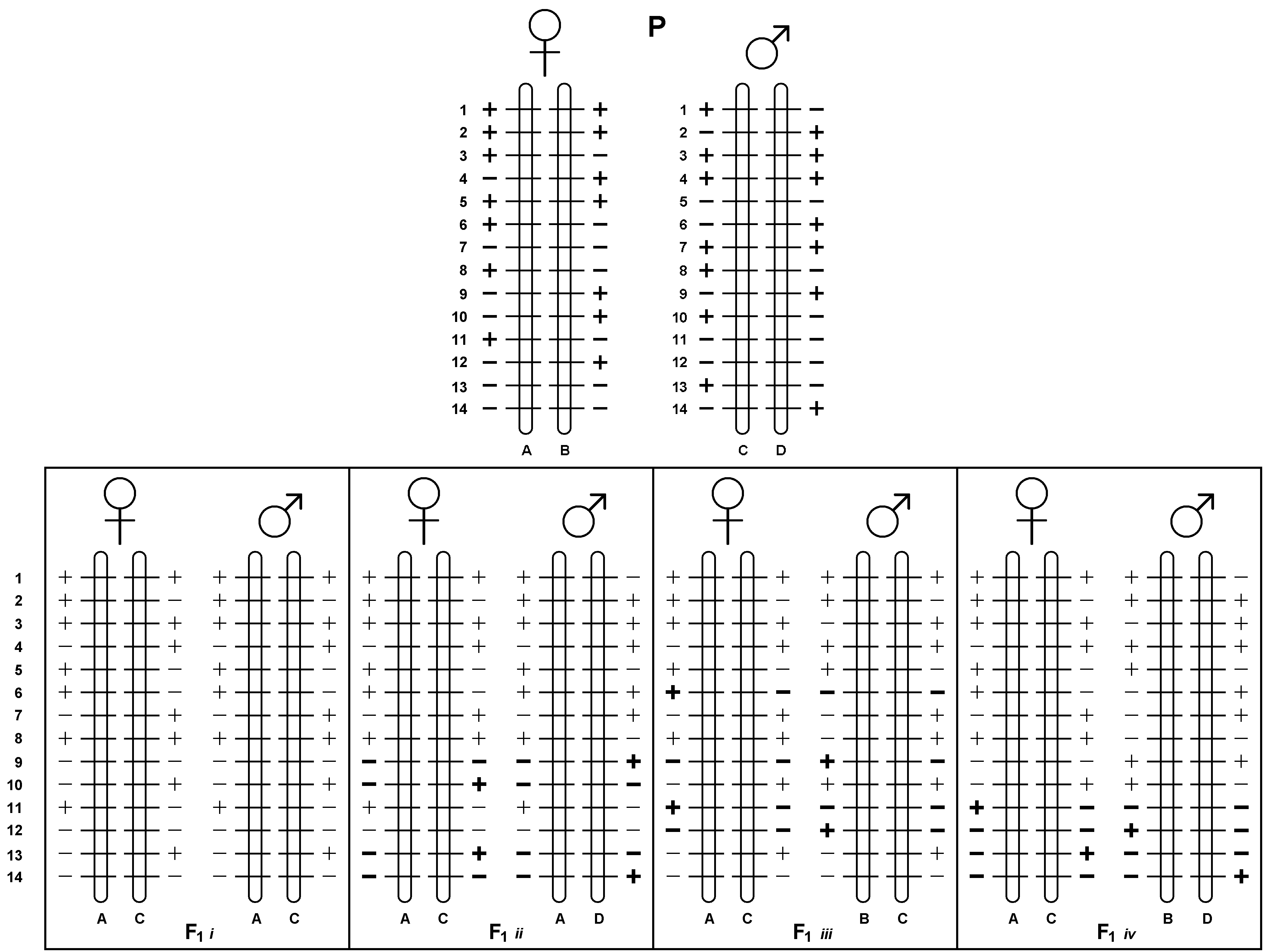


Fig. 10. Marker associations in P- and F_1_ generation of an outbred full-sib cross. The P-generation has four different chromosomes (A, B, C, D). The fourteen loci on these chromosomes cover all the genotype combinations that are potentially informative in the offspring (“+” = dominant allele, “–” = recessive). FI and MI markers are in bold, (the unmarked loci are uninformative allele combinations and BI markers). F_1_ *i* has no FI or MI markers, while the other combinations have two of each. Recombination is not included in this scheme.

chromosome and different paternal chromosomes, *iii* F_1_ male and female with the same paternal chromosome and different maternal chromosomes, *iv* F_1_ male and female with four different chromosomes. These four types of F_1_ combinations are shown in Fig. 10 and 11, which illustrate the effects of a full-sib cross on the 1:1 and 3:1 segregating markers respectively.

When the male and female have the same chromosome combination, there are no MI or FI markers available (Fig. 10 F_1_ *i*). However, MI and FI markers can (but not necessarily do) occur if the P-male chromosomes recombine within the region in which the markers are located before they are inherited by the F_1_ male. Thus a consequence of this full-sib design is that there is a deficiency of FI and MI markers in 25% of the linkage groups, and chromosome prints cannot be determined directly for them without FI marker information available. The chromosome prints for these groups must therefore be reconstructed in an alternative way as described in Supplement 2. With one chromosome in common (i.e type *ii* and *iii*), FI and MI markers do occur, but their linkage phases are all identical as can be seen in Fig. 10, where the “+” signals are restricted to one of the chromosomes in both individuals.


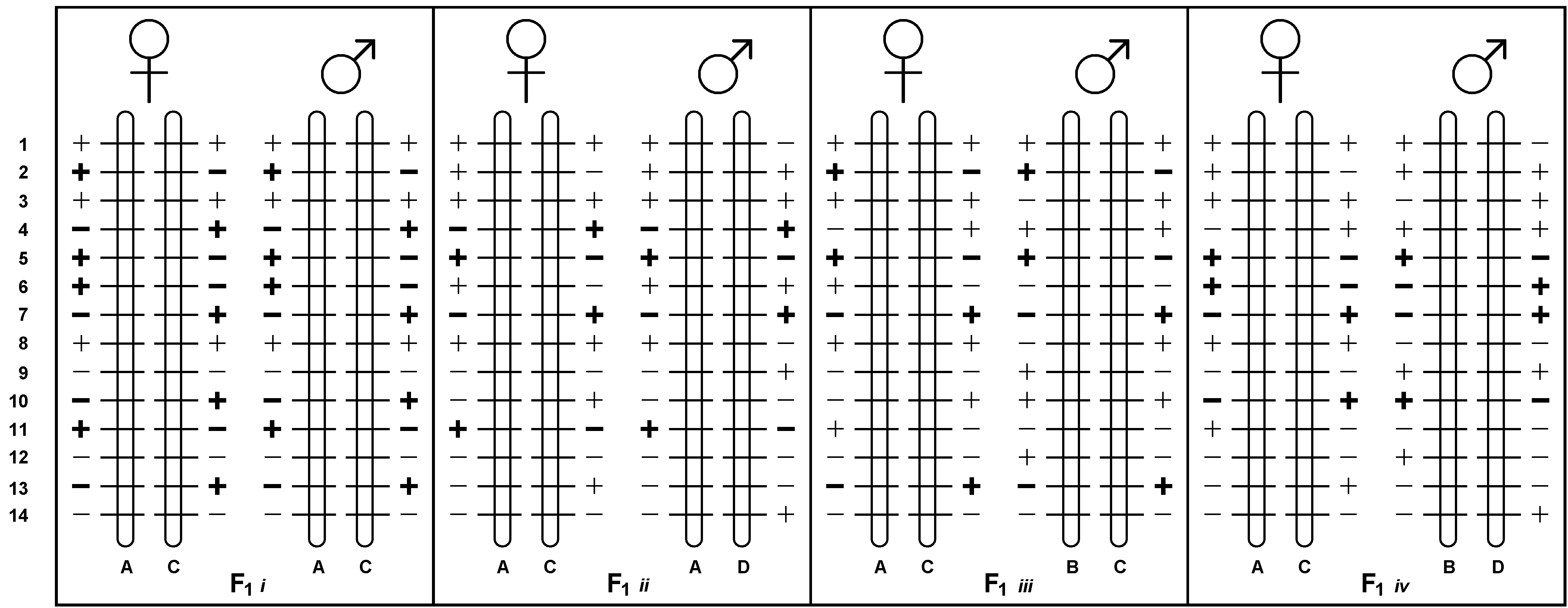


Fig. 11. Same F_1_ combinations as in Fig. 10, but now with only BI markers highlighted. Combinations *i*, *ii* and *iii* have marker combinations in fixed phases (i.e. “+–/+–” or “–+/–+”). F_1_ *iv* has independent marker associations (i.e. “+–/+–”; “–+/–+”;“+–/–+”;“–+/+–”).

The presence of chromosomes that are identical by descent in both F_1_ male and female (i.e. type *i*, *ii*, *iii*) results in informative[[1]](#footnote-2) BI markers with exclusively peakpresents or exclusively peakabsents within an F_2_ individual if the chromosome that was inherited from the F_1_ male is non-recombinant[[2]](#footnote-3). This is demonstrated in Fig. 11, where maternal and paternal syntenic BI marker values (i.e. + or –) are either exclusively identical or exclusively opposite (in *i*, *ii*, *iii*). E.g., If an F_2_ individual inherits the “A” chromosome of the F_1_ female from the type *ii* combination in Fig. 11, markers 4 and 7 are informative because they are peakabsent on that chromosome. The same F_2_ individual inherits either the “A” or the “D” chromosome from the F_1_ male. In case of the “A” chromosome, all markers (4 and 7) are peakabsent, whereas the “D” chromosome gives exclusively peakpresents for these loci. This example is representative for all chromosome combinations in the F_2_ offspring (of type *i*, *ii* and *iii* crosses).

Recombination (in the F_1_ male) results in a mix of both peakpresent and peakabsent BI markers within an F_2_ individual. The linkage groups with four different chromosomes in the F_1_ (type *iv*) have no restrictions on BI marker combinations.

1. “Informative” BI markers are those that remain after censoring, i.e. those that inherited a peakabsent from the F_1_ female and thus have only a male-informative component. [↑](#footnote-ref-2)
2. Or a F_1_ male inherited chromosome that is at least non-recombinant within the region for which markers are available. The female chromosome is obligatory non-recombinant. [↑](#footnote-ref-3)
